# Coastal Supra‐Permafrost Aquifers of the Arctic and Their Significant Groundwater, Carbon, and Nitrogen Fluxes

**DOI:** 10.1029/2024GL109142

**Published:** 2024-11-21

**Authors:** Cansu Demir, James W. McClelland, Emily Bristol, Matthew A. Charette, M. Bayani Cardenas

**Affiliations:** ^1^ Department of Earth and Planetary Sciences The University of Texas at Austin Austin TX USA; ^2^ Marine Biological Laboratory The Ecosystems Center Woods Hole MA USA; ^3^ The University of Texas at Austin Marine Science Institute Port Aransas TX USA; ^4^ Woods Hole Oceanographic Institution Marine Chemistry and Geochemistry Woods Hole MA USA

**Keywords:** submarine groundwater discharge, coastal Arctic lagoons, Cryohydrogeology, groundwater‐surface water interactions, permafrost

## Abstract

Fresh submarine groundwater discharge (FSGD) can deliver significant fluxes of water and solutes from land to sea. In the Arctic, which accounts for ∼34% of coastlines globally, direct observations and knowledge of FSGD are scarce. Through integration of observations and process‐based models, we found that regardless of ice‐bonded permafrost depth at the shore, summer SGD flow dynamics along portions of the Beaufort Sea coast of Alaska are similar to those in lower latitudes. Calculated summer FSGD fluxes in the Arctic are generally higher relative to low latitudes. The FSGD organic carbon and nitrogen fluxes are likely larger than summer riverine input. The FSGD also has very high CO_2_ making it a potentially significant source of inorganic carbon. Thus, the biogeochemistry of Arctic coastal waters is potentially influenced by groundwater inputs during summer. These water and solute fluxes will likely increase as coastal permafrost across the Arctic thaws.

## Key Points

- Summer fresh submarine groundwater discharge (FSGD) to the Alaskan Beaufort Sea is only 3%–7% of rivers but carries as much organic matter
- Summer FSGD delivers a median of 116 (interquartile range: 35–405) and 6 (2–21) kg/d per km dissolved organic carbon and nitrogen
- Fresh groundwater at the beach of Simpson Lagoon (SL) has a median PCO_2_ of ∼33,000 μatm implying substantial CO_2_ flux

## 1 Introduction

Submarine groundwater discharge (SGD) is important for coastal biogeochemistry and ecosystem health, as it is a major source of organic matter and nutrients (Santos et al., 2021). The flow and mixing of fresh and saline SGD creates subterranean estuaries (STEs), hotspots for biogeochemical reactions (Harris et al., 2017; Lecher, 2017; Moore, 2010; Santos et al., 2008). Fresh submarine groundwater discharge (FSGD) into Arctic coastal waters is also crucial for coastal water budgets (Harris et al., 2017). Investigations of SGD and STEs have mainly been in lower‐latitude systems, and knowledge in the Arctic remains limited (Lecher, 2017).

In the Arctic, FSGD originates and flows in the seasonally‐thawed shallow active layer (0.2–1 m thick) (Nelson et al., 1998; Wales et al., 2020). This layer, also called a supra‐permafrost aquifer, may continue beyond beaches or coastal bluffs into coastal waters, providing paths for groundwater flow into unfrozen coastal sediment overlying submarine permafrost (Charkin et al., 2017; Dimova et al., 2015; Lecher, 2017). SGD is controlled by the ice‐bonded permafrost configuration, which hinders and directs water movement (Lecher, 2017). Coastal permafrost varies in extent (Angelopoulos et al., 2019; Kasprzak, 2020; Overduin et al., 2012; Pedrazas et al., 2020; Swarzenski et al., 2016). Ice‐free sediment beneath coastal waters forms primarily due to salt‐induced freezing point depression (Osterkamp & Harrison, 1977) and heat supplied by the overlying water (van Everdingen, 2005). Thus, while permafrost may be ubiquitous in the Arctic, many factors also favor the existence of STEs.

Groundwater in the Arctic has high concentrations of dissolved organic carbon and nitrogen (DOC, DON) (Connolly et al., 2020), and potentially carbon dioxide (CO_2_) from DOC respiration. When it enters coastal waters, CO_2_‐rich groundwater may make surface waters a CO_2_ source to the atmosphere (Wang et al., 2018) and cause acidification (Cardenas et al., 2020; Liu et al., 2023). Moreover, DON from groundwater may enhance coastal primary production (Bronk et al., 2007). Therefore, quantifying FSGD solute fluxes along the Arctic coast is crucial.

The few SGD studies from the Beaufort Sea coast of Alaska were limited to total SGD, the sum of fresh and recirculated saline SGD (TSGD = FSGD + RSGD). These studies utilized an indirect mass balance approach with radium and radon tracers measured over hours to a few days (Bullock et al., 2024; Connolly et al., 2020; Dimova et al., 2015; Lecher et al., 2016). Aside from our related efforts (Guimond et al., 2023), there have been no published direct observations of coastal groundwater flow in the Arctic. Moreover, the rates and fluxes of DOC, DON, CO_2_, and DIC in FSGD have not been quantified using direct hydrologic and geochemical measurements and observation‐informed flow and transport mechanistic models. This study aims to: (a) Provide insights into the summer (July–September) hydraulic, thermal, and geochemical regime of Arctic STEs; and (b) constrain FSGD fluid and solute fluxes.

## 2 Methods

During summer in the Arctic, the depth of the coastal ice table can range from decimeters to a few meters (Dimova et al., 2015; McCann & Hannell, 1971; Owens & Harper, 1977; Pedrazas et al., 2020; Sobota et al., 2018). Depths to ice‐bonded permafrost of 10–20 m within 500 m from shore are common (Angelopoulos et al., 2019; Overduin et al., 2012, 2016), but depths of <5 and >20 m are also possible (Overduin et al., 2016; Pedrazas et al., 2020).

**Figure 1:**
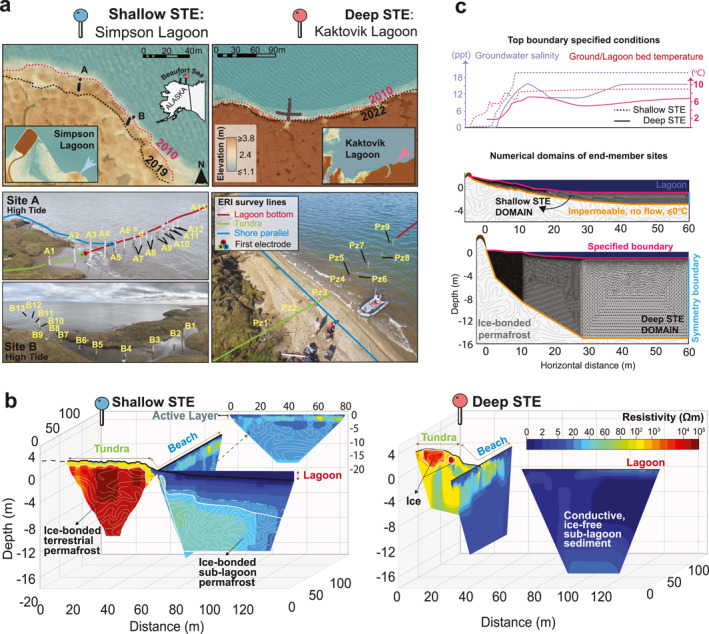
(a) Field sites (Simpson Lagoon (SL)‐A/B and Kaktovik Lagoon (KL)) given with piezometer IDs. (b) Inverted Electrical resistivity imaging (ERI) surveys, conducted in this study for SL, combined with previously reported surveys for KL (Pedrazas et al., 2020). (c) Numerical domains, conceptualized based on ERI observations, with assigned boundary conditions. Hydraulic head follows the topography until the shoreline, after where it is equal to the lagoon level.

**Figure 2:**
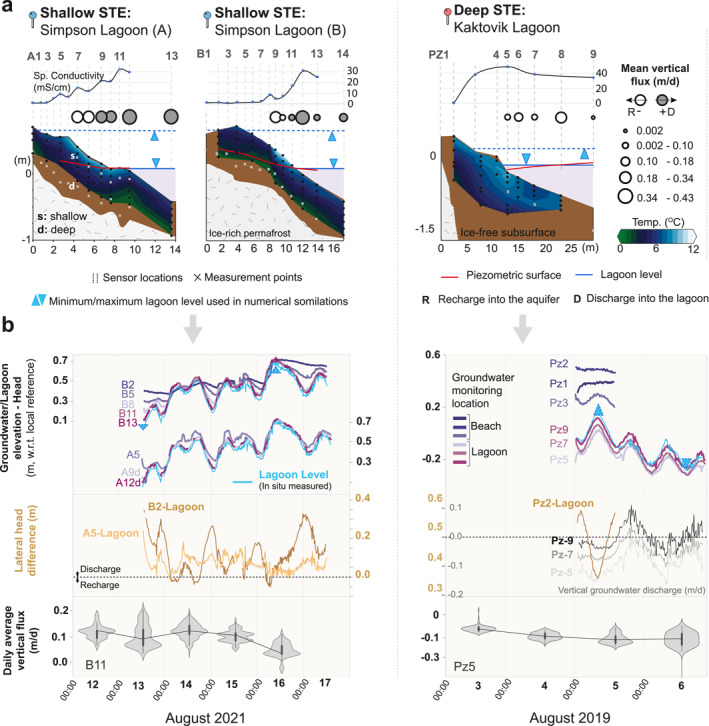
(a) Probed ice‐bonded permafrost table, temperature profiles during low lagoon level periods, time‐snapshot salinity profiles, and in situ average vertical groundwater fluxes. (b) Lagoon and groundwater level time series, estimated lateral hydraulic head differences, and in situ daily average vertical groundwater fluxes (see Figure S3c in Supporting Information S1 for all locations) at the Shallow‐STE and Deep‐STE sites.

We established observational transects at two sites with shallow and deep ice tables. The end‐member STE sites are at the coast of Kaktovik Lagoon (KL, “Deep‐STE site”) and Simpson Lagoon (SL, “Shallow‐STE site”) bordered by barrier islands in the Beaufort Sea of Alaska (Figure 1a). The field sites represented the low‐lying Arctic coast of the Alaskan North Slope, characterized by high‐centered polygonal tundra transitioning into lagoons with a continuous topographical gradient. Sites with a beach were selected to capture a wider supra‐permafrost aquifer extent. At both sites, we manually mapped the ice table, installed multi‐depth soil temperature probes from the beach to the subtidal zone, installed piezometer transects equipped with water level, salinity, and temperature probes (Figures 1a and 2a), and sampled groundwater. Electrical resistivity imaging (ERI) surveys indirectly mapped the ice table in three perpendicular directions in SL site A (Figure 1a): (a) Lagoon bottom, (b) Shore parallel, and (c) Tundra (Survey and inversion details in Text S2, Figures S1, S2, and Table S1 in Supporting Information S1). In KL, areas captured with ERI were similar to that of SL (Pedrazas et al., 2020). Aquifer saturated hydraulic conductivity (*K*) was estimated using sediment samples analyzed with laboratory constant head tests and empirical approaches based on grain size distributions (Text S4 in Supporting Information S1).

We quantified groundwater fluxes using complementary approaches. Thermal profiles were interpreted with analytical solutions of the 1‐D steady‐state heat transport equation to calculate vertical groundwater fluxes, following the methodology of Bredehoeft and Papadopulos (1965) (Figure S3b in Supporting Information S1). Profiles within the saturated inter‐subtidal areas were selected to validate the vertical‐flow‐only assumption of the method (Taniguchi et al., 2003) and sensors located >20 cm below seabed were chosen, when available, to avoid interference of shallower diel signals (Kurylyk et al., 2017) (Figure S4 in Supporting Information S1). Vertical hydraulic head gradients between lagoon and piezometric groundwater level were used for point calculations via Darcy's Law (Figure S3a in Supporting Information S1). Curve matching of the above two methods provided *K* estimates (Figures S3c, and S4 in Supporting Information S1). Point‐based heat‐tracing‐derived fluxes were integrated over areas with positive seepage to find in situ estimates of FSGD (Figure S5 in Supporting Information S1). The most comprehensive of the approaches is the calculation of FSGD using numerical groundwater flow and transport model ensembles. The models couple and solve equations for non‐isothermal, density‐driven saturated groundwater flow, salt, and heat transport (equations and parameters: Tables S2, and S3 in Supporting Information S1). Electrical resistivity imaging and hydro‐thermal observations constrained the finite‐element model domains and boundary conditions (Figures 1b and 1c). A steady‐state thermohaline system was assumed during summer season, neglecting daily changes from atmospheric, oceanic, and terrestrial forces. Dynamic surface water levels, resulting in RSGD due to salinity gradients, and tidal and wave pumping (Smith, 2004) were not simulated. Instead, the following steady lagoon boundary conditions were used: (a) The lowest measured level (representing strong terrestrial forcing), (b) the average level, and (c) the highest measured level (representing weak terrestrial forcing). FSGD was calculated by integrating discharge over the top model boundary where groundwater flux is positive/upward. An ensemble of models was implemented for each aquifer archetype (the 3 lagoon levels × 100 *K* cases). This approach enabled understanding of flow and transport behavior under varying *K* and tidal stages, and isolation of FSGD from TSGD. To cover a broader range of possible STE configurations between the two end‐members, ensemble model inputs were varied through random sampling (Monte Carlo), quantifying uncertainty (mostly representing natural variability) of the observations. The ensemble model outputs were compared and synthesized with in situ estimates.

Groundwater samples collected along piezometer transects in KL (August 2019, *n*_samples_ = 20) and SL (August 2021, *n*_samples_ = 30) were analyzed for DOC/N concentrations (C_DOC_, C_DON_). The KL DOC/N data set was merged with that sampled (*n*_samples_ = 20) in 2014–15 in KL by Connolly et al. (2020). This combined C_DOC/N_ data set for KL better approximates the spatial and temporal variability along the lagoon. In addition, in situ measured pH and partial pressure of CO_2_ in groundwater from SL (*n*_samples_ = 13; Figure S6 in Supporting Information S1) were incorporated into equilibrium equations to estimate dissolved inorganic carbon concentrations (C_CO2(aq)_, C_DIC_) (See Text S6, and Table S4 in Supporting Information S1). We separated the C_DOC_, C_DON_, C_CO2(aq)_, and C_DIC_ data sets of KL and SL into two distributions ‐ fresh (5,000 μS cm^−1^) and saline (20,000 μS cm^−1^) groundwater. FSGD‐driven DOC, DON, CO_2(aq)_, and DIC mass fluxes were quantified by multiplying their fresh water statistical concentration distributions by the ensemble FSGD distributions (Cabral et al., 2023). Additional details of our approaches are summarized in Figure S7 in Supporting Information S1.

## 3 Results

### 3.1 Key Characteristics of the Two End‐Member Coastal Arctic Aquifers

Simpson Lagoon and Kaktovik Lagoon are reasonable shallow and deep end‐members given the very limited field observations throughout the Arctic. Ice‐bonded permafrost typically appears with resistivity values of 10^3^–10^6^ Ω‐m in inverted ER tomograms from on‐land surveys and of >30 Ω‐m in underwater surveys (Kasprzak, 2020; Pedrazas et al., 2020). In the Shallow‐STE site (SL), the ice‐bonded permafrost was more prominent and ERI detected its top (∼10^3^ Ω‐m contour line) at depths of ≤1 m in the tundra and of 1–3 m in the first 15 m in the beach‐lagoon interface (Figure 1b). The unfrozen sediment reached 7 m in thickness as the frost table continued and gradually dipped into the seabed further into the lagoon. At the Deep‐STE site (KL), no ice was detected within the top few meters of the beach or lagoon bed through ground probing (Pedrazas et al., 2020), except for the abruptly declining ice table present near the tundra along the first few meters of the transect (>1.5 m drop within the first 4 m; Figure 2a). Previous ERI surveys showed sporadic ice‐bonded permafrost laterally and vertically along the transect, and an unfrozen aquifer up to 20 m deep at the beach (Pedrazas et al., 2020).

In Shallow and Deep‐STE sites, *K* varied significantly based on a synthesis of 53 *K* estimates determined via various techniques (see Section 4, and Text 4 in Supporting Information S1). An empirical distribution of *K* (Median(*M*):16.1 m day^−1^; Mean ($\bar{X}$):46.6, Standard deviation(σ):126.1; Interquartile range (*IQR*):9.1–46.8) was fitted with a lognormal distribution ($\bar{X}$:47.51, σ:131.7). Generated random values from fitted distributions were used as input in numerical models (Figure S8 in Supporting Information S1). In this study, the reported values for mean ($\bar{X}$ and μ_x_) and standard deviation (σ and σ_x_) are for lognormal and normal distributions, respectively.

### 3.2 Coastal Groundwater Dynamics: In Situ Observations

Expectedly, the STEs were cold at both sites (Figure 2a) with temperatures decreasing with depth until the ice table. The summer subsurface temperatures were generally well above freezing, ranging between 0.9–10.8°C and 4.8–13.6°C at the Shallow‐STE and Deep‐STE sites, respectively. Both aquifers were mostly in stable thermal stratification (Figure 2a).

Daily average astronomical tidal range was approximately 0.2 m for both sites in August (Figure S9 in Supporting Information S1). Our observations coincided with surges, with rising and falling phases in SL and KL, respectively. At each site, tidal signal in groundwater decreased landward. In KL, the furthest inland piezometers (Pz1 and 2 in Figure 1a) showed minimal tidal influence due to a wider beach; unlike SL with a narrower beach (Figure 2). The gradient of decreasing hydraulic head and increasing salinity from tundra to lagoons are direct evidence for FSGD. The steady positive hydraulic head difference between inland end‐member piezometer (Pz‐2 and B2) and lagoon level, suggesting persistent flow toward lagoons, was higher in KL ($\mu_{x}$:0.45 m) compared to SL ($\mu_{x}$:0.12 m).

### 3.3 Groundwater Fluxes

**Figure 3:**
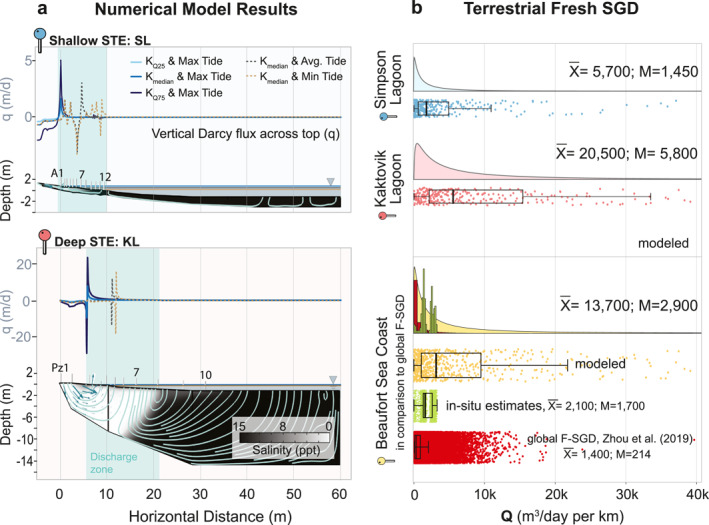
(a) Numerical flow and transport model outputs for the Shallow‐STE and Deep‐STE sites (high lagoon level scenario). (b) Ensemble‐derived fresh submarine groundwater discharge statistical distributions (empirical histograms and/or fitted distributions) compared with in situ estimates and global estimates from Zhou et al. (2019).

At both sites, field observations and model outputs showed that fresh terrestrial groundwater meets recirculated saline groundwater at a nearshore mixing zone. This confirms the presence of STEs in Arctic lagoon coasts (Figures 2a and 3a for beach‐lagoon salinity profiles). The models suggest a larger freshwater‐saltwater interface at the Shallow‐STE site than at the Deep‐STE site. An upper‐saline circulation cell was present at the beach of the Deep‐STE site, possibly due to saline seawater infiltrating‐exfiltrating during a past surge event (Figure 3a and Figure S9 in Supporting Information S1).

SGD occurred in the vicinity of the shoreline at both sites (Figure 3a). During the highest observed meteorological tide with surge, the modeled discharge zone (+, upward flux) was 8 m wide at the Shallow‐STE, and 14 m wide at the Deep‐STE sites. During the lowest observed tide, the discharge zone extended seaward; reaching up to ∼10 and 19 m at the Shallow and Deep‐STE sites, respectively.

The spatially integrated median FSGD flux estimates for the ensemble of models for SL and KL were 1,450 and 5,790 m^3^ day^−1^ km^−1^, respectively (Statistics in Table S5 in Supporting Information S1; Figure 3b). The Deep‐STE site had higher FSGD than the Shallow‐STE site due to higher mean terrestrial hydraulic head gradient during the study period. Combining the model ensembles of the Shallow and Deep‐STE sites resulted in an overall median of 2,900 m^3^ day^−1^ km^−1^. Extrapolation and integration of the overall FSGD distribution to the Alaskan Beaufort Sea coast (1,957 km coastal length) gave a median of 5.7 × 10^6^ m^3^ day^−1^ in late summer.

In situ techniques revealed daily vertical groundwater flux, manifesting variations in magnitude and direction within shallow depths along transects and among sites (Figure 2a, and Figure S3c in Supporting Information S1). Absolute mean vertical flux within the inter‐to‐subtidal zone during the measurement period varied between 0.002 and 0.4 m day^−1^ in both sites. The flow patterns were mostly consistent with lateral gradient‐driven discharge, that of seawater recirculation within the inter‐tidal zone indicated by vertical salinity gradients (Table S6 in Supporting Information S1), and with flow and transport model outputs (Figure 3a).

Spatial integration of in situ local FSGD estimates over the discharge zone (Figure S5 in Supporting Information S1) gave a median of 2,230 m^3^ day^−1^ km^−1^ at the Shallow‐STE site and 320 m^3^ day^−1^ km^−1^ at the Deep‐STE site (Statistics in Table S7 in Supporting Information S1). The combined distribution resulted in a median FSGD of 1,700 m^3^ day^−1^ km^−1^.

### 3.4 Estimation of Dissolved Carbon and Nitrogen Fluxes

**Figure 4:**
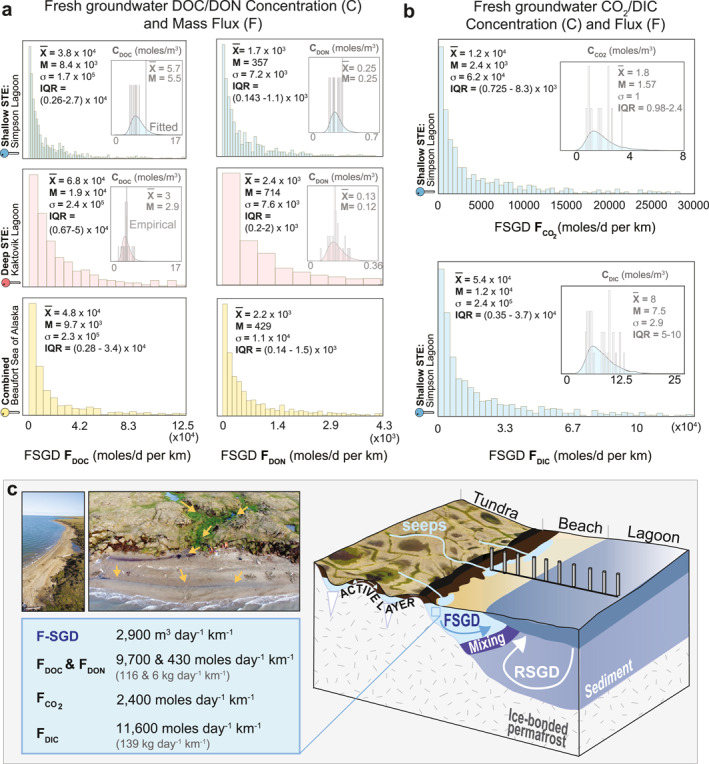
(a) Fresh submarine groundwater discharge derived DOC/N, (b) CO_2_, and DIC concentration and mass flux distributions (c) Arctic STE: conceptualization, summary median fluxes into Beaufort Sea, aerial images (by Nathan Sonderman), August 2019, Kaktovik Lagon; arrows indicate channelized surface flow becoming submarine groundwater discharge.

At the Deep‐STE site, distributions of fresh groundwater C_DOC_ and C_DON_ had medians of 2.9 and 0.14 mol m^−3^, respectively (Statistics in Table S7 in Supporting Information S1; Figure 4a). At the Shallow‐STE site, they were 5.6 and 0.25 mol m^−3^, respectively. The concentrations at the Shallow‐STE site were almost double of those at the Deep‐STE site. The combined distribution of saline groundwater C_DOC_ and C_DON_ of both sites had medians of 1.2 and 0.057 mol m^−3^, respectively ($\bar{X}$:2.4 and 0.13; σ:4.2 and 0.29; *IQR*:0.417–3.75 and 0.02–0.14 mol m^−3^).

At the Deep‐STE site (KL), the FSGD‐derived mass flux of DOC (F_DOC_) and DON (F_DON_) had medians of 1.9 × 10^4^ and 714 mol day^−1^ km^−1^ (225 and 10 kg day^−1^ km^−1^), respectively. The *IQR* for mass fluxes on the high end were up to an order of magnitude higher than the values reported previously for KL, which were 14–71 and 1–4 kg day^−1^ km^−1^ for F_DOC_ and F_DON_, respectively (Connolly et al., 2020). Therefore, in addition to the FSGD, F_DOC_ and F_DON_ turn out to be higher than previously reported when sources of uncertainty (ensemble results) are considered. At the Shallow‐STE site, F_DOC_ and F_DON_ had medians of 8.4 × 10^3^ and 360 mol day^−1^ km^−1^ (101 and 5 kg day^−1^ km^−1^), respectively. We estimated higher F_DOC_ and F_DON_ for the Deep‐STE site due to its higher FSGD despite higher C_DOC/N_ in the Shallow‐STE site samples. If the combined flux distribution of Shallow‐STE and Deep‐STE sites is representative of the broader region, then the median F_DOC_ and F_DON_ to the Beaufort Sea are 9.7 × 10^3^ and 430 mol day^−1^ km^−1^ (116 and 6 kg day^−1^ km^−1^), respectively.

The partial pressure of CO_2_ (P_CO2_) in fresh groundwater (<5,000 μS/cm) at 50–70 cm depths at the beach measured in an additional trip to the Shallow‐STE site in July 2023 had a median of 32,872 μatm ($\bar{X}$:38,527; σ:23,550; *IQR*s:19,497–52,409 μatm; n_sample_:10; Table S8 in Supporting Information S1). Calculated C_CO2(aq)_ and C_DIC_ were *M*:1.57 mol m^−3^ and *M*:7.4 mol m^−3^, respectively (Figure 4b, Table S7 in Supporting Information S1), which includes the uncertainty in pH measurements (Table S9 in Supporting Information S1). Thus, FSGD delivers a median of 2,400 mol CO_2_ d^−1^ km^−1^ and 11,600 mol DIC d^−1^ km^−1^, respectively.

## 4 Discussion and Conclusions

To independently test and validate our FSGD estimates, we estimated the possible amount of water (PAW) available for flow using the following simple water balance over a narrow coastal contributing area (150 km^2^, or ∼200 m wide strip of land from the shore along Beaufort Sea coast, Figure S10a in Supporting Information S1) throughout the spring and summer:

PAW=ALW+P‐ET

where ALW is water stored in the active layer from the previous year assuming full saturation, and P and ET are total precipitation and evapotranspiration from June to September (See Text 7 for details).

The resulting PAW estimate of 1.7 × 10^7^–1.6 × 10^8^ m^3^ is similar in magnitude as our total FSGD estimate (3.4 × 10^8^ m^3^) for summer (∼60 days). However, the active layer of previous fall may not be fully saturated, especially in high‐centered polygonal tundra with steep micro‐topographical gradients (Liljedahl, Hinzman, & Schulla, 2012), leading to lower ALW than estimated. Our modeled FSGD estimates (3.4 × 10^8^ m^3^ over 60 days) to the Beaufort Sea (1,957 km) are in the high end likely due to model limitations. Nonetheless, our in situ FSGD estimates (2 × 10^8^ m^3^ along Beaufort Sea over 60 days) and those from previous studies (0.5–2.5 × 10^8^ m^3^ over 60 days and 1,957 km coastline (Connolly et al., 2020)) confirm the order of magnitude of modeled FSGD over the 60‐day summer period. The similarity in PAW and FSGD estimates suggests that most runoff flowing in the channelized polygonal tundra within the contributing area likely discharges ultimately as FSGD (Figure 4c).

The September‐to‐end‐of‐June discharge from the largest rivers in the North Slope, the Sagavanirktok (0.9–2.5 km^3^ yr^−1^), Kuparuk (0.6–1.6 km^3^ yr^−1^), and Colville Rivers (12.2–27.7 km^3^ yr^−1^), are respectively 65%, 63%, and 62% of the total annual discharge, with the vast majority occurring during the spring (April) freshet. The remaining 35%, 37%, and 38% occur in summer (McClelland et al., 2014). Based on this, our total FSGD (median) estimate is 3%–7% of the combined summer river discharge of 8.6–20 × 10^7^ m^3^ day^−1^ (July–September, ∼60 days; see Text 7, Table S10 in Supporting Information S1).

The sum of summer (July and August) riverine solute fluxes for the three rivers are 3.12–7.08 × 10^5^ kg DOC day^−1^ and 1.43–3.3 × 10^4^ kg DON day^−1^ (data is from McClelland et al. (2014), See Text S7, Table S10 in Supporting Information S1). A comparison of our summer FSGD DOC/N mass flux *IQR* estimates (0.7–7.9 × 10^5^ kg DOC day^−1^ and 0.39–4.11 × 10^4^ kg DON day^−1^, over 1957 km‐long Beaufort Sea coastline) with the ranges reported for rivers shows that FSGD can deliver as much or even more DOC/N as the major rivers in summer. Alaskan rivers deliver more water and DOC/N in spring peak discharge period (May–June) compared to the remaining hydrologic year (McClelland et al., 2014). The comparisons made above are only for the 60‐day summer. The summer period estimations indicate that FSGD‐derived DOC/N input to the Beaufort Sea (*IQR*:4.2–47.4 × 10^6^ kg DOC and 2.3–24.7 × 10^5^ kg DON) can be up to 43% and 63% of the mean combined annual river DOC and DON inputs, respectively (1.1–1.6 × 10^8^ kg DOC and 3.9–4.6 × 10^6^ kg DON from (McClelland et al., 2014)). Though Monte Carlo‐based FSGD‐derived solute estimates include spatial variability at the beach, any DOC/N consumption or production within STEs will modify the final flux into the lagoons.

The PCO_2_ in fresh beach groundwater in SL was at the high end for unpolluted groundwater globally (Macpherson, 2009). Summertime soil PCO_2_ in polygonal tundra at a drained lake basin in Utqiaġvik, Alaska was similarly high (>10,000 μatm at 15–20 cm depths), reaching up to 100,000 μatm during freeze‐up in mid‐November (Wilkman et al., 2021). Additionally, a geometric mean of 1.5 mol CO_2_(aq) m^−3^ was reported for the same site at a different time (Lipson et al., 2012). These values are similar to our fresh groundwater CO_2_ measurements ($\mu_{x}:$ 1.8 mol m^−3^, Table S8 in Supporting Information S1), confirming high CO_2(aq)_ delivered via FSGD to the Arctic coast.

Our study has key limitations. Our flux estimates cannot be extrapolated for the whole year because the active layer is mostly frozen. We expect lower amounts of FSGD during spring river peak discharge (May–June) and freeze‐up periods (after September) due to a lack of liquid water in the active layer, and increasing RSGD values from thawing to open‐water period due to increasing wave energy, frequent wind/storm events, and density (salinity/temperature) instabilities. We hypothesize that as the thaw season progresses, groundwater inputs become increasingly significant compared to river inputs.

The gradually deepening shallow aquifer in SL resembles submarine permafrost aquifers along large inlet lagoon (Overduin et al., 2012) and non‐lagoon (Angelopoulos et al., 2019) low‐lying Arctic coasts. Thus, SL may better represent the majority of the Alaskan Beaufort Sea coast. The deeper ice‐bonded permafrost table (thicker aquifer) at KL could be due to its depositional environment (Text S1 in Supporting Information S1) or an inherited older talik (Pedrazas et al., 2020). While we captured two potential end‐member sites with sloping beaches, other coastal parts vary, including cliffs, no lagoons, broader flats, and degraded and submerged ice‐wedge polygon tundra. Future studies are needed to resolve potential uncertainty due to spatial and temporal variability in SGD.

Our modeled (M:2,900 m^3^ day^−1^ km^−1^) and in situ (M:1,700 m^3^ day^−1^ km^−1^) summer estimates indicate that Arctic coastal aquifers can deliver as much or perhaps even more FSGD as lower latitude coastal aquifers (M:214 m^3^ day^−1^ km^−1^ by Zhou et al. (2019), and Figure 3b, also see TSGD comparison between Arctic lagoon (ranging in magnitude of 10^4^–10^5^ m^3^ day^−1^ km^−1^) versus Lower‐latitude seas and lagoons (10^3^–10^5^ m^3^ day^−1^ km^−1^) in Text S7, Table S11 in Supporting Information S1). The findings emphasize the importance of SGD in conveying organic matter, inorganic carbon, and potentially other solutes to Arctic lagoons.

## Conflict of Interest

The authors declare no conflicts of interest relevant to this study.

## Supporting information

Supporting Information S1

## Data Availability

Numerical simulations were conducted in COMSOL Multiphysics® 6.0. Field data, COMSOL numerical model files (average water level case), and data produced in this study are publicly available in Demir et al. (2024).
