# Supplementary material for: Coastal Supra‐Permafrost Aquifers of the Arctic and Their Significant Groundwater, Carbon, and Nitrogen Fluxes
Source: Geophys Res Lett. 2024 Nov 21;51(22):e2024GL109142. doi: 10.1029/2024GL109142 (PMC11579974; doi:10.1029/2024GL109142)
Supplement: Supplementary file 1 — Supporting Information S1 [file GRL-51-0-s001.pdf]

Geophysical Research Letters

Supporting Information for

Coastal supra-permafrost aquifers of the Arctic and their significant groundwater, carbon, and nitrogen fluxes

Cansu Demir[1], James W. McClelland[2], Emily Bristol[3], Matthew A. Charette[4], and M. Bayani Cardenas[1]

[1] The University of Texas at Austin, Department of Earth and Planetary Sciences, Austin, Texas, USA.

[2] Marine Biological Laboratory, The Ecosystems Center, Woods Hole, Massachusetts, USA.

[3] The University of Texas at Austin, Marine Science Institute, Port Aransas, Texas, USA.

[4] Woods Hole Oceanographic Institution, Marine Chemistry and Geochemistry, Woods Hole, Massachusetts, USA.

Corresponding author: Cansu Demir; (cdemir@utexas.edu)

Contents of this file

Text SI.1 to SI.7
Figures S1 to S10
Tables S1 to S11

Supplementary Information (SI) to the methods and results presented in the main text

SI.1. Further details about field sites

In the Simpson Lagoon (SL) sites, degraded blocks of tundra and buried peat within the intertidal and shallow subtidal zones indicate that it is an actively eroding system. This contrasts with Kaktovik Lagoon (KL), where deposition seems to be overpowering or keeping up with erosion and therefore the beaches are wider. Arctic lagoons are diverse and reflect different impacts from terrestrial and oceanic forces. At SL (Deep STE Site), barrier islands have wider, permanently open tidal inlets which promote larger quantities of water moving between the lagoon and the Beaufort Sea during the open-water season. This can promote more dramatic wave action, storm surge and/or high tides (Bird, 1994). Therefore, SL exhibits characteristics more typical of open marine coasts while KL is more of a low-energy estuarine system (Emery & Stevenson, 1957).

SI.2. Previous electrical resistivity imaging surveys at Kaktovik Lagoon and new surveys and analyses details for Simpson Lagoon transects

We used a SuperSting R8 eight-channel ERI equipment from Advance Geosciences Inc. (AGI). The survey lines consisted of tundra, shore parallel and lagoon bottom transects as shown in Figure 1 in the main text. Land cables were used for the 55 m-long tundra transect with 56 electrodes and spacing set to 1 m. Underwater cables (56 electrodes) with 1.5 m electrode spacing were laid along the 82.5 m-long shore parallel and lagoon bottom transects. Each transect was double-surveyed with Dipole-Dipole (D-D) and Schlumberger (S) array settings done back-to-back. Using AGI EarthImager™ 2D software, we merged the apparent resistivity outputs of D-D and S surveys. Inversions were implemented on the merged datasets for tundra and shore parallel transects (with RES2DINV® software). However, due to high error in the inversion of lagoon bottom merged data, we used only the Schlumberger array survey outputs for inversion of this transect. The lagoon bottom survey was inverted using the underwater inversion option in AGI EarthImager™ 2D with measured lagoon resistivity of 0.67 Ω−m. Inversion parameters are given in Table S1.

The panels given in Figures S1-2 compare the inverted resistivity outputs (inverted tomograms) of each transect with the relative model sensitivity values calculated during the inversions. The sensitivity function estimates how much the potential measured by the array is affected by changes in resistivity. Sensitivity is higher when the subsurface has a greater influence on the measurements and exponentially decreases with depth (Areas that are closer to the electrodes have higher sensitivity and often higher model resolution). In our surveys, most of the features that we are interested in (ice-rich permafrost table, frozen/unfrozen pockets, conductive material due to salt water saturation) show up in the first few meters of the transects where model sensitivity

is the highest. This allows for more reliable interpretation.

In KL, we probed the ice table from the edge of the tundra towards the shore with a push-point metal rod. At the first point, the ice table was detected at 28 cm below the ground surface and it declined abruptly (~1.5 m drop over 4 m in lateral distance) (main text, Fig. 2a). On the other hand, in SL, we defined the ice table at the depth at which the PVC piezometers cannot be pushed further (~1 m). However, the ERI survey along the shore, in Fig. S1a, indicates at least 2 different layers of different characteristics. This top aquifer is ~1m thick, saturated with fresh to saline water along the cable length, and is the layer where our beach piezometers are in. This top layer is sporadically (laterally) confined by a ground-proofed ice-bonded thin sediment layer (mostly peat) and below that we see the freshwater-saturated resistive pockets (> 29.5 Ohm-m) as the second layer. The conductive lens separated by an ice-bonded layer from the above fresh-water saturated pockets might suggest the presence of a possible third, deeper thawed layer (thin saline aquifer).

SI.3. Measurements of hydraulic head, water level, salinity, and temperature along transects

All in situ measurements were done along temporarily installed piezometer transects at each site. The first piezometers of shore-perpendicular transects were located at the interface of the tundra and beach, capturing the fresh groundwater component. In SL, we installed two shore-perpendicular transects (A, 9.5 m and B, 14 m, Fig.1a, 2a) of partially screened 1.25" diameter PVC piezometers (n_A=18, n_B=13), approximately 42 m away from each other. All piezometers were pushed to the ice-bonded permafrost table, except six of them at transect A, which were installed to a shallower depth next to their pairs to capture vertical gradients of groundwater head and salinity. In KL, a shore-perpendicular (~25 m long) and a crossing shore-parallel (~40 m long) transects were installed. The latter was used only for water sampling purposes. At this site, unlike SL, piezometers were not pushed to the frozen ground, due to deeper ice-depths. The limiting factors for the piezometer installment depth were sediment resistance and strength of the PVC material. In KL, beach piezometers were selected to be fully screened to capture phreatic surface. The ones installed in the seabed were partially screened, at both sites.

Measurements of groundwater and lagoon level and salinity were made using pressure transducers of various models and manufacturers (InSitu® Aquatrolls, Leveltrolls, Rugged Trolls and Solinst® LT/LTC Leveloggers; LTC: Level, Temperature, Conductivity) and electronic water level meters. The piezometers which were installed with transducers are A5, A9s (LTC), A9d, A12, B2, B5, B8, B11, B13, Pz1, Pz2 (LTC), Pz3 (LTC), Pz5 (LTC), Pz7 (LTC), Pz9 (LTC). The lagoon levels (temperature and salinity) were monitored with LTC sensors attached to the casing of lagoon piezometers, near the

seabed. Continuous measurements were recorded during August 5 - 7, 2019 in KL, and August 13 to 17, 2021 in SL. In Simpson Lagoon, groundwater salinity was not continuously monitored with sensors due to lack of transducers with integrated salinity sensors. However, we used a handheld pH and conductivity sensor (Myron® Ultrameter™) to record the spatial specific conductance variation along and among the transects (Fig. 2a). All the transducer pressure measurements were corrected with respect to air pressure (data from Barter Island airport weather station, and a Barologger® installed at our site in SL). All level and elevation measurements were cross-referenced, and tied to each other with a laser theodolite in KL and a robotic total station (Trimble S6 Robotic Total Station) in SL.

Along the piezometer transects, we installed multi-depth temperature sensors (HOBO 4-channel loggers and Alpha Mach Trods) into the sediment/seabed to capture the spatial distribution of temperatures in the beach and intertidal zone (Fig. 2a), as well as to estimate groundwater fluxes using heat as a tracer. These were located at A1, 3, 5, 7, 8, 9, 10, 12, 13, and B2, 4, 6, 8, 9, 10, 11, 12, 13, 14 at the SL sites, and PZ2, 4, 5, 6, 7 at the KL site. Sensor depth intervals varied from location to location, ranging from 10 to 21.6 cm. Continuous measurements of level and temperature were recorded for the duration of field campaigns.

Lagoon level data recorded during and before our measurement period by NOAA, https://tidesandcurrents.noaa.gov/ (for SL, station: Prudhoe Bay), and the Beaufort Lagoon Ecosystems (BLE) LTER program (for KL) (Beaufort Lagoon Ecosystems LTER, 2020) were retrieved (Fig. S9), in addition to our measurements in the field. According to these measurements and weather data from © WeatherSpark.com, both systems are under the influence of a westerly wind driven positive surge during the measurement period (i.e. the difference between measured and predicted astronomical tides in SL and KL are positive). The westerly wind events occurred between Aug 15 – 23 in SL (dies down on the 21th for a day) and Aug 01 – 02, 04 – 05 in KL. They caused a rise in water levels, which, in SL, inundated the beach completely and even overtopped the polygonal trough next to the narrow beach at Site B (main text, Fig. 1). In KL, the start of our measurements coincided with peaking of water levels due to the surge, then it fell down during the monitoring period. Long term lagoon salinity measurements in KL, showed correlation with water levels. When there is positive surge (measured>predicted), salinity increases, and when it is the opposite, salinity decreases.

SI.4. Estimation of submarine groundwater discharge using field-based approaches

Two in situ methods were employed to estimate SGD to the lagoons: (1) temperature-depth measurements in the sediment and their analysis with theoretical models of coupled heat and groundwater flow, and (2) vertical hydraulic head gradients at the seabed (Darcy's Law method) (Fig. S3a, S3b). For both of these techniques to be

applicable, the location of interest should be fully saturated and fluxes should be negligible in the horizontal direction (Kurylyk et al., 2019). Our models and the literature show that the groundwater flux vector (q, m.day^{-1}) is predominantly vertical at the inter- to subtidal areas where most of the total SGD discharge occurs (Taniguchi et al., 2003). Therefore, six and four of thermistor-piezometer-pair locations in the lagoon were selected at site SL and KL, respectively, following the criteria above (Fig. S4).

As groundwater flows, it advects heat and thus affects the subsurface temperature distributions via conduction, assuming local thermal equilibrium. This is the premise for Method (1). Here we employed the analytical solution of the steady-state 1-D heat transport equation to calculate vertical groundwater fluxes by following the methodology explained in ref. (Bredehoeft & Papaopulos, 1965). In shallow surface water systems, periodic diel seabed temperature signals are present but these dampen at shallow depths (typically a decimeter) which helps make the steady-state assumption valid beyond several centimeters deep in the sediment (Bredehoeft & Papaopulos, 1965; Kurylyk et al., 2017). Therefore, to limit the influence of diel signals on the flux estimations, we selected sensors located >20 cm below seabed, when available (exceptions (>10 cm) were the thermistors located next to Pz6, Pz7 and Pz9, due to limited depths of measurements.) (Fig. S4, 0m represents the lagoon-sediment interface). The diel temperature zone range gets smaller with seaward distance due to the insulation provided by the thicker lagoon water layer. We automated FLUX_LM (an Excel spreadsheet developed by ref. (Kurylyk et al., 2017) for estimating vertical groundwater flux using the Bredehoeft and Papadopulos theoretical method (ref. (Bredehoeft & Papaopulos, 1965))) with a Python code for implementing flux calculations at different time intervals (5 min) for the duration of the temperature measurements. Figure S3b provides the thermal parameters used in the estimates. Due to the assumption that the system is at steady-state at any given time, we estimated numerous F-SGD values over a period but only reported the daily mean values. The results can be reproduced using the code and the data published on GitHub (https://github.com/cannsudemir/Auto-FLUX-LM).

Continuous lagoon and piezometric groundwater level measurements allowed for estimation of vertical hydraulic gradients between the seabed and the piezometric measurement point ~20-55 cm in the seabed (Method 2, Fig. S3a). The flow directions indicated by vertical head gradients helped cross-check the co-located temperature profiles (Recharge: concave up, discharge: convex up), since vertical heterogeneity might change the direction of flow and result in variations in the curvature of the temperature-depth profile (Kurylyk et al., 2017). This double-method approach allowed us to estimate saturated hydraulic conductivity (K) at given locations by adjusting (optimizing) K values until the curves of Method 1 and 2 show a reasonable overlap. Results from both in situ observation-based methods (Method 1 & 2) were compared (Fig. S3c).

In addition to the curve-fitting approach via Method 1 and 2, K was estimated

through laboratory methods, and empirical equations. Sediment samples collected from discrete locations in KL were analyzed with a CAMSIZER® particle size analyzer. The estimated grain size distribution parameters were used to solve empirical equations from Beyer (1964); Carman (1937), (1956); Hazen (1892); Kozeny (1927), (1953); and Terzaghi & Peck (1964) to calculate the saturated *K* of various sediment types, assuming fresh water density and viscosity. Undisturbed sediment samples collected in SL were analyzed with constant head tests (Darcy's Law) using KSAT® device (Meter Environment), and saturated *K*s were estimated. Overall, from both sites, we collected a total of 53 *K* measurements (Fig. S8). The *K* information were used to determine inputs for groundwater flow models (see SI.5 for details).

We plotted the location specific estimated vertical fluxes ($m.d^{-1}$) over the lateral distance along the transects (Fig. S5a), then, calculated the positive area under the flux-distance curve for each moment (Fig. S5b) to estimate the total FSGD discharge ($m^3.day^{-1}.km^{-1}$).

Although it is very practical to implement in the field, like many other field-based (in situ) techniques, there are several limitations to the heat tracing method for groundwater flux estimations. By assuming vertical homogeneity in thermal properties, steady-state conditions, and the absence of lateral flux nearshore, the method may be underestimating the fluxes. In addition, there is a limit on how far into the lagoon measurements can be made to cover the whole seepage area. This spatial bias may also result in underestimation of groundwater discharge values. A previous study suggested that the heat tracing method can be insensitive to tidally driven recirculation of groundwater, unlike measurements of discharge with a seepage meter which measures both recirculated and fresh groundwater discharge (Taniguchi et al., 2003). While this may indicate that our estimations only count for FSGD, which we are mainly interested in, we cannot disregard the possible contribution of recirculated SGD in our estimations. Partly due to the reasons given above, we conducted numerical flow and transport simulations to base FSGD estimations solely on terrestrial and quasi-steady state density gradients, and in order to consider the uncertainty in hydraulic conductivity (*K*).

SI.5. Estimation of submarine groundwater discharge using numerical groundwater flow and transport models

The flowchart given in Figure S7 demonstrates our methodology in estimating fluxes with numerical modeling and in situ observational techniques. The details of the model building steps and our assumptions are given below.

The hypothetical domains for SL and KL coastal aquifers (Fig. 1c) were simulated in a finite-element, Multiphysics numerical modeling environment (COMSOL Multiphysics®). The models solve the coupled equations for non-isothermal, density-

driven groundwater flow in saturated conditions (Eqn. 3-4 in Table S2), salt transport (Eqn. 5) and heat transport (Eqn. 6). These equations are coupled through fluid density, the parameter $\boldsymbol{\rho_w}$ in Eqn. 7. The parameters used in the model runs are provided in Table S3.

In situ and ex-situ observations were used to constrain and design the conceptual models for both sites. The domain geometries were identified based on the observations of topography, and ice-table depth surveyed via Electrical Resistivity Imaging surveys. At the Deep STE Site of KL, the maximum depth of measurement with 1.5 m electrode spacing and dipole-dipole arrays was ~15 m where ice-bonded permafrost was still absent (Pedrazas et al., 2020). On the other hand, as described in the main text, at the Shallow STE Site of SL, a shallow ice table was at detectable depths (1-7 m) based on combined dipole-dipole and Schlumberger arrays (electrode spacing: 1 and 1.5 m for on land and underwater surveys, respectively (See SI.2. for SL ERI survey details). Based on these findings, we assigned hypothetical bottom no-flow boundary conditions mimicking the ice-table, which starts deepening towards offshore and stays at a constant depth after a distance >25 m into the lagoon (7 m and 15 m depths for SL and KL, respectively). Although ERI detected an unfrozen bulb of sediment beneath the tundra side in the KL transect, based on our observations, we assumed that fresh groundwater flow will mostly occur in the active layer and recharge the beach aquifer through the drainage of surface channels. Therefore, the conductive-unfrozen portion below the tundra was not included in the aquifer domain of the model for KL. The right boundary is extended sufficiently seawards (≥60 m) in order to go far beyond the expected extent of lateral flow and so that this boundary can be assigned as a symmetry (no-flow) boundary (Eqn. 8, Table S2). The top boundary conditions were assigned with selected snapshots of the field-measured dynamic ground temperature, and salt concentration (Fig. S1a). Hydraulic head followed the topography until the shoreline, after where it is equal to lagoon level.

One of the main assumptions of the models is that the system is at steady-state during summer season (open-water), neglecting the daily changes due to changes in atmospheric, oceanic and terrestrial forces. With this in hand, we aim to focus solely on snapshots of this steady system and understand the flow and transport behavior of the hypothetical system with real boundary conditions under different hydraulic conductivity and tidal stage scenarios. Therefore, to be clear, dynamic surface water levels were not explicitly simulated. Rather snapshots of different tidal stages were used as steady boundary conditions: (1) minimum observed lagoon water level, when the terrestrial forcing is strongest, (2) average or mid-level, and (3) maximum water level, when the terrestrial forcing is weakest. In coastal settings, TSGD is composed of fresh SGD (FSGD) and recirculated seawater (RSGD). RSGD is driven by density (salinity) gradients, tidal

pumping, wave pumping and wave setup (Smith, 2004). Because of the quasi steady-state assumption for simulating non-dynamic tides, resulting in minimal salinity (density) gradients, our models only estimate for a small portion of recirculated seawater discharge (RSGD) which comes solely from steady salinity gradients. Therefore, FSGD pertains to the portion of the model boundary where the groundwater flux is positive/upwards.

The steady-state distributions of temperature-salt concentration-head was achieved by a long-term run (~100 years) with the given boundary conditions. However, it should be noted that this does not mean the system needs 100 years to reach steady-state. Our model started with almost 0 ppt salinity and hydrostatic conditions, which is not representative of the beginning of each summer season. Therefore, in reality, reaching quasi-steady-state after a real-world perturbation like changes in water level likely happens much faster (over a few hours to several days). Using outputs of the steady-state model as an initial condition, a suite of numerical models was implemented for varying K and lagoon level values.

Empirical distribution (ED) of K values (n=53, see SI.4. for details) were fitted with a log-normal distribution (FD). We randomly sampled 100 values (creating the RSD) from the FD to use as inputs for the numerical models (implemented in MATLAB). This Monte Carlo approach represents the natural variability and quantifies its contribution to uncertainty. Statistical descriptions for each ED, FD, and RSD were compared and found to be very similar, illustrating that the RSD is a good representative of the ED (Fig.S8). For each lagoon coast aquifer archetype, 3×100 different cases were simulated. Each model tests three distinct lagoon water stages (minimum, average, and maximum of the range measured during our period of observation) and every level scenario is simulated with 100 different unique randomly sampled K values, which in turn produce distributions of possible groundwater flux values (Fig. 3). Finally, the estimated vertical groundwater flowrates in m.day^{-1} were then integrated over the discharge zones to find the groundwater fluxes in m^{3}.day^{-1}.km^{-1} (Table S5).

SI.6. Groundwater sampling and estimation of constituent fluxes

In August 2019 and 2021, we collected 20 and 40 groundwater samples extracted from each piezometer along two transects at the KL site (shore parallel and perpendicular), and one shore-perpendicular transect at the SL site via peristaltic pumps, respectively. We filtered the pumped water with 0.45 μm in-line capsule filters (Geotech high-capacity dispos-a-filter™) or PES filters. The samples were stored in acid-washed polycarbonate bottles and kept refrigerated (Simpson Lagoon samples were kept frozen at -20°C.) until analyzed for concentrations of dissolved organic carbon (C_{DOC}) and total dissolved nitrogen (C_{TDN}) in Shimadzu TOC-V CSH analyzer and TBN-1 total nitrogen detector at the University of Texas Marine Sciences Institute (UTMSI). Dissolved organic

nitrogen (DON) was calculated by subtracting dissolved inorganic nitrogen (DIN) concentrations from total dissolved nitrogen (TDN). DIN concentrations – the sum of nitrate, nitrite, and ammonium – were determined using continuous flow-analyzers Lachat Quick Chem 8500 at UTMSI (2019 data) and FIAlyzer at the Virginia Institute of Marine Science (2021 data). The KL DOC/N concentrations gathered in 2019 were merged with chemical observations from 2014 and 2015 sampling campaigns in KL (Connolly et al., 2020).

During an additional trip to SL on July 30 and 31, 2023, we measured the gas phase mole fraction (wetCO_2, ppm_v) of CO_2 in 13 groundwater and 2 nearshore lagoon samples in situ. Groundwater was pumped through a semi-permeable membrane-based CO_2 sensor (CO_2-Pro™ CV by Pro-Oceanus, factory-calibrated to a range of 0 – 20,000 ppm_v or μatm with accuracy of around 20 μatm) with attached gas-impermeable tubing via a peristaltic pump (see Fig. S6 for the picture of the in-situ measurement setup). The membrane-based CO_2 sensors measure the gas phase mole fraction (wCO2, ppm_v) of CO_2 that is in equilibrium with surrounding liquid by allowing the diffusion of gas (CO_2) from the liquid across the membrane to a gaseous headspace where the gas analyzed with a non-dispersive infrared sensor (more details can be found at https://pro-oceanus.com). During the measurement process, water was pumped through the gas-equilibration head chamber for a minimum 15 minutes to reach equilibrium across the diffusion membrane. The reading at the end of this period was recorded together with a simultaneous measurement of sample temperature, pH and salinity (Myron® Ultrameter™). Partial pressure of CO_2 was estimated by P_{CO2}=wetCO_2 x P_{wet}, where P_{wet} is the total pressure of the internal gas which includes water vapor pressure and assumed to be equal to 1 atm. Equilibrium dissolved CO_2 concentration was calculated by Henry's Law ($CO_{2(aq)}$=f(CO_2) x K_0) with fugacity (f(CO_2)) correction. The equilibrium constant K_0 was adjusted as a function of in-situ measured sample temperature and salinity (Table S8) according to the formula given by Weiss (1974). Calculations of P_{CO2} (μatm), $CO_{2(aq)}$, and other components of the carbonate system (HCO_3^-, CO_3^{2-}; DIC, Alkalinity) were done following the equilibrium equations given in Table S4 using 'seacarb', an R package (Gattuso et al., n.d.). Equilibrium constants K_1 and K_2 were also adjusted as a function of in-situ measured sample temperature and salinity according to the formula given by (Millero, 2010). P_{CO2} decreased with salinity and pH (Fig. S6). Nearshore lagoon water P_{CO2} was measured twice over two consecutive days as 835 – 974 ppm_v (~**μatm**), which is higher than the average atmospheric P_{CO2} on the same days measured in Barrow, AK as ~410 ppm_v. While this hints at the importance of lagoon water CO_2 on water-atmosphere fluxes, more data points need to be collected to make conclusions on the source/sink state of the nearshore lagoon.

The fresh groundwater C_{DOC}, C_{DON}, $C_{CO2(aq)}$, and C_{DIC} empirical datasets of different sizes, as well as the fresh groundwater fluxes for KL and SL (n=100 each, model

estimated), were fitted with lognormal distributions and randomly sampled to match a common distribution size with n=700. Then, the distributions of concentrations and randomly sampled fresh groundwater fluxes were multiplied to estimate the FSGD derived DOC – DON – $CO_{2(aq)}$ – DIC mass fluxes (Table S7; $C_{CO2(aq)}$, and C_{DIC} values are given in Table S8.). Since $CO_{2(aq)}$ – DIC samples were specific to Simpson Lagoon only, the concentrations were multiplied by the water fluxes of SL. At the end of each day when we measured P_{CO2}, the pH of a pH=7 standard (buffer) was measured to determine drift and sensitivity/error, which we found to be ±0.05. The effect of this uncertainty on DIC concentrations were accounted for in the final FSGD DIC mass flux distributions (Table S9).

SI.7. FSGD in comparison to lower latitude coasts, terrestrial storage – recharge, and river discharge

We compiled a table of SGD values estimated for various mid-low latitude coastal systems and made estimations (when not given) to calculate total SGD in $m^3.day^{-1}.km^{-1}$ by using the coastline length for each coastal system (Table S11). In addition, we compared our values to the global FSGD dataset estimated by (Zhou et al., 2019), which excludes polar regions (Fig. 3b).

Our FSGD estimate for the entire Beaufort Sea coast (1957 km long) is M= 6×10^6 $m^3.day^{-1}$ ($\bar{\mathbf{X}}$: 2.6×107; σ: 1.1×108; *IQR*: 0.21 – 1.9×107 $m^3.day^{-1}$). Assuming that this representative flux is constant and continuous through the summer (July to September, 60 days), we calculated the total water discharge as 3.6×10^8 m^3. This terrestrially driven fresh groundwater is sourced from gradual thawing of the active layer, melt of snow accumulated during the winter, and precipitation (rain + snow) occurring during the Spring – Summer period. However, most of the snowmelt occurs during April-May and only a small portion melts in June (Fig. S10d). Snow melt exits the land as surface runoff before the active layer is barely thawing in June and therefore, it does not change the active layer water balance and is neglected. We made back-of-the-envelope calculations for estimating possible amount of water (PAW) available for flow (surface runoff + groundwater) over a narrow (~200 m wide) contributing area (A, 150 km^2) along the Beaufort Sea coast (Fig. S10a). We arbitrarily chose a width of 200 m because observations indicate that the groundwater draining into the beach is water that has drained into and though the troughs of the ice wedge polygons. These troughs typically form a network draining a few polygons which are several meters to tens of meters (up to 30 m) wide.

A simple water budget for the coastal strip of land is given by:

$$PAW = ALW + P - ET$$

where ALW is water stored in the active layer from the previous year, P and ET are total precipitation and evapotranspiration from June to September.

The active layer thickness (ALT), i.e., the maximum depth of thaw, varies between 0.2 m and 1 m in the coastal plain of Alaska (Nelson et al., 1998). Our late summer field observations indicated that the coastal tundra active layer consists of acrotelm (live peat and vegetation) and catotelm (old, dead peat) whose porosity (ϕ) varies between 0.78 and 0.98 (Chen et al., 2020). Based on these ϕ ranges, we estimated that 2.3×10^7 – 1.5×10^8 m^3 of water (ALT × Area × ϕ) is stored in the active layer and available for flow from spring to the end of summer (June-September). Our assumptions for this estimation consist of: (1) specific yield in the active layer is high, (2) the active layer was fully ice saturated before seasonal melting, and (3) a major portion of surface run-off eventually becomes groundwater at the tundra-beach interface and discharges as SGD. To calculate the net precipitation (P-ET), the accumulated area-averaged ET (Fig. S10c), for the coastal area from June 1 to August 31 in 2019 retrieved from GLDAS CLSM (CLSM025_DA1_D v2.2, (Li et al., 2020)) is subtracted from area averaged accumulated P for the same duration retrieved from GPM IMERG (Final Precipitation L3 1 day 0.1° × 0.1° V06, (Huffman, G.J., Stocker, E.F., Bolvin, D.T., Nelkin, E.J., 2019), Fig. S10b). The resultant PAW is 1.7×10^7 – 1.6×10^8 m^3 for a 150 km^2 contributing area (Snowmelt over June 2019 -retrieved from GLDAS Model GLDAS_CLSM025_DA1_D v2.2- is estimated as 1.3×10^5 m^3 over the 150 km^2 area, and is negligible with respect to our PAW estimates). This amount (June-September; 90 days) is the same magnitude as our FSGD estimate (July-September; 60 days). Our FSGD estimates are only valid for the summer period of approximately 60 days, however, even with the additional limited amount of FSGD added during the spring period, order-of-magnitude-wise, our FSGD estimates are consistent with water available for flow.

For river vs. groundwater DOM flux comparisons, we used the information provided in McClelland et al. (2014) for three largest rivers in the North Slope (Sagavanirktok, Kuparuk, and Colville) draining into Beaufort Sea (Table S10) (McClelland et al., 2014). We based our comparisons on the summer river discharge and DOM flux values (after peak discharge period, July – September, ~60 days). Given the information that water discharge from September to the end of peak discharge period, late June, is 65%, 63%, 62% of the total annual discharge (0.9 – 2.5, 0.6 – 1.6, 12.2 – 27.7 $km^3.yr^{-1}$) from Sagavanirktok, Kuparuk, and Colville rivers, respectively, 35, 37 and 38% of total annual river discharge occurs during the summer period (McClelland et al., 2014). With this information, we estimated summer time riverine water discharge and DOM mass fluxes, and compared them with the FSGD and FSGD derived DOM mass fluxes.

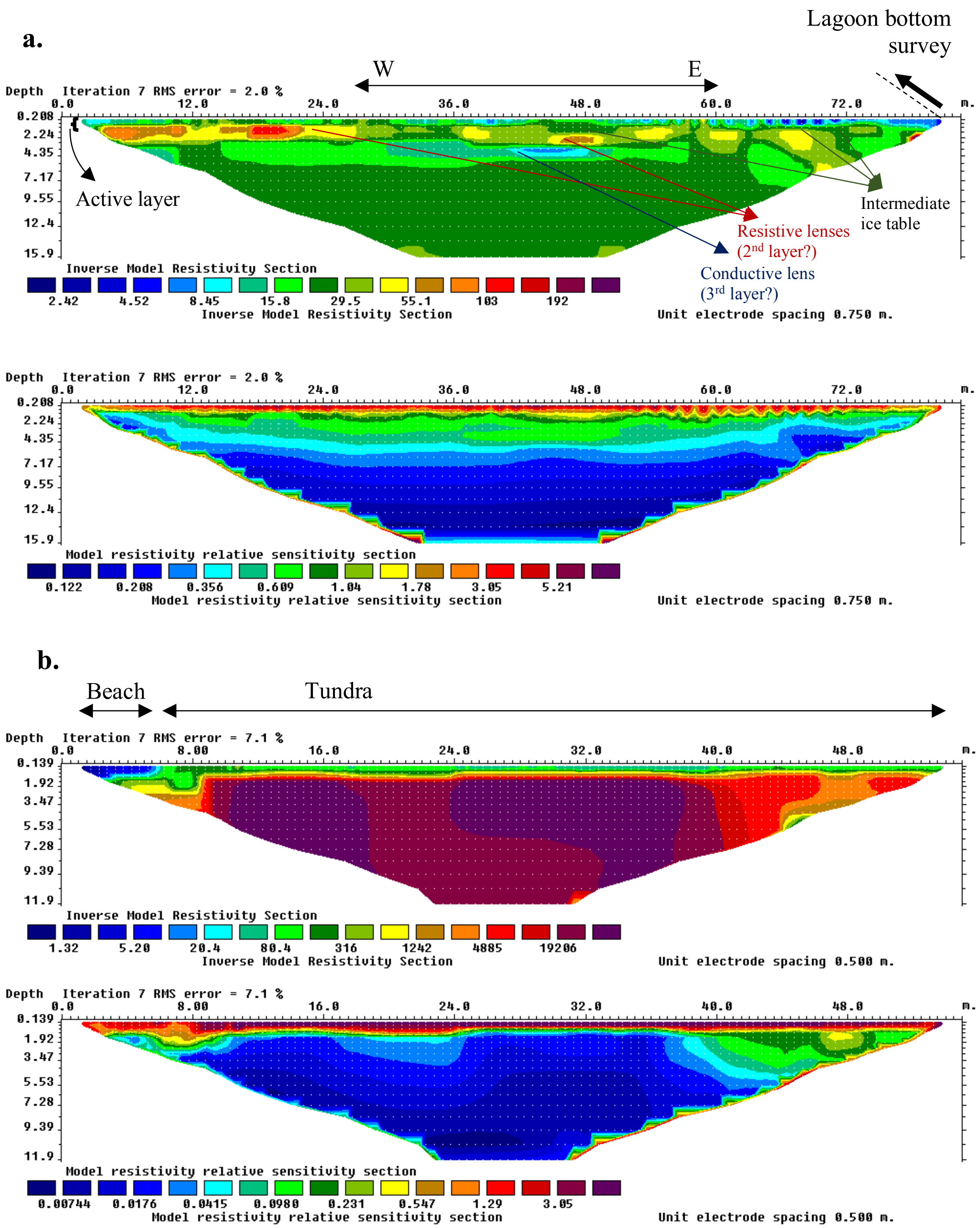

Figure S1. Inverted ER fields and sensitivity fields for (a) shore parallel, and (b) tundra (on-land) surveys. Topography is not included.

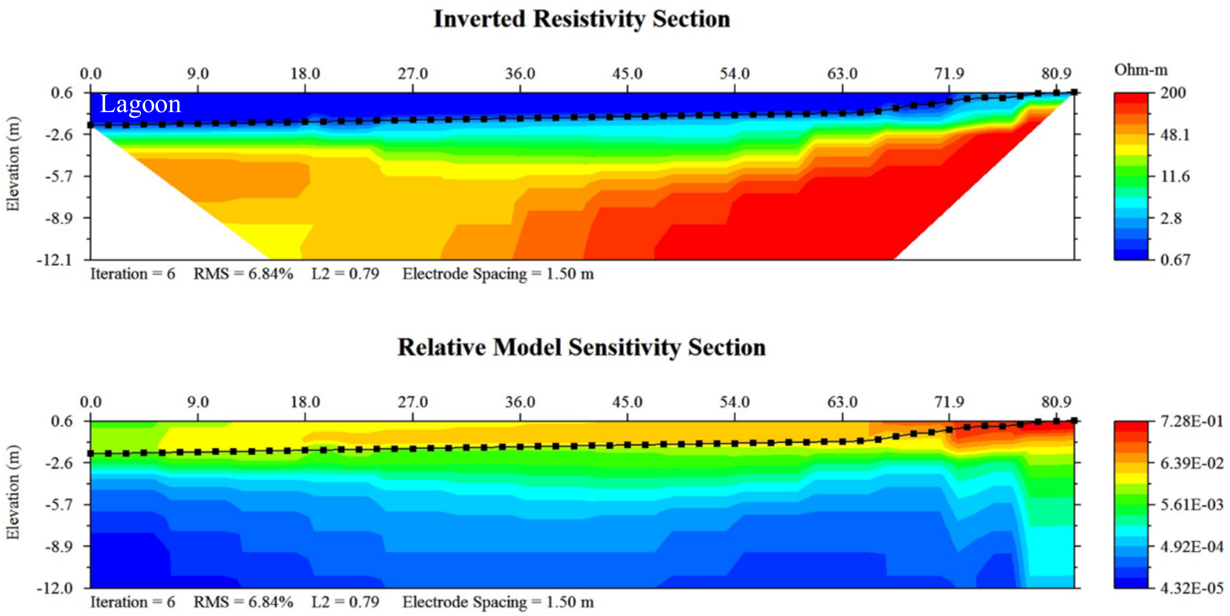

Figure S2. Inverted ER fields and sensitivity fields for the lagoon bottom survey (underwater survey).

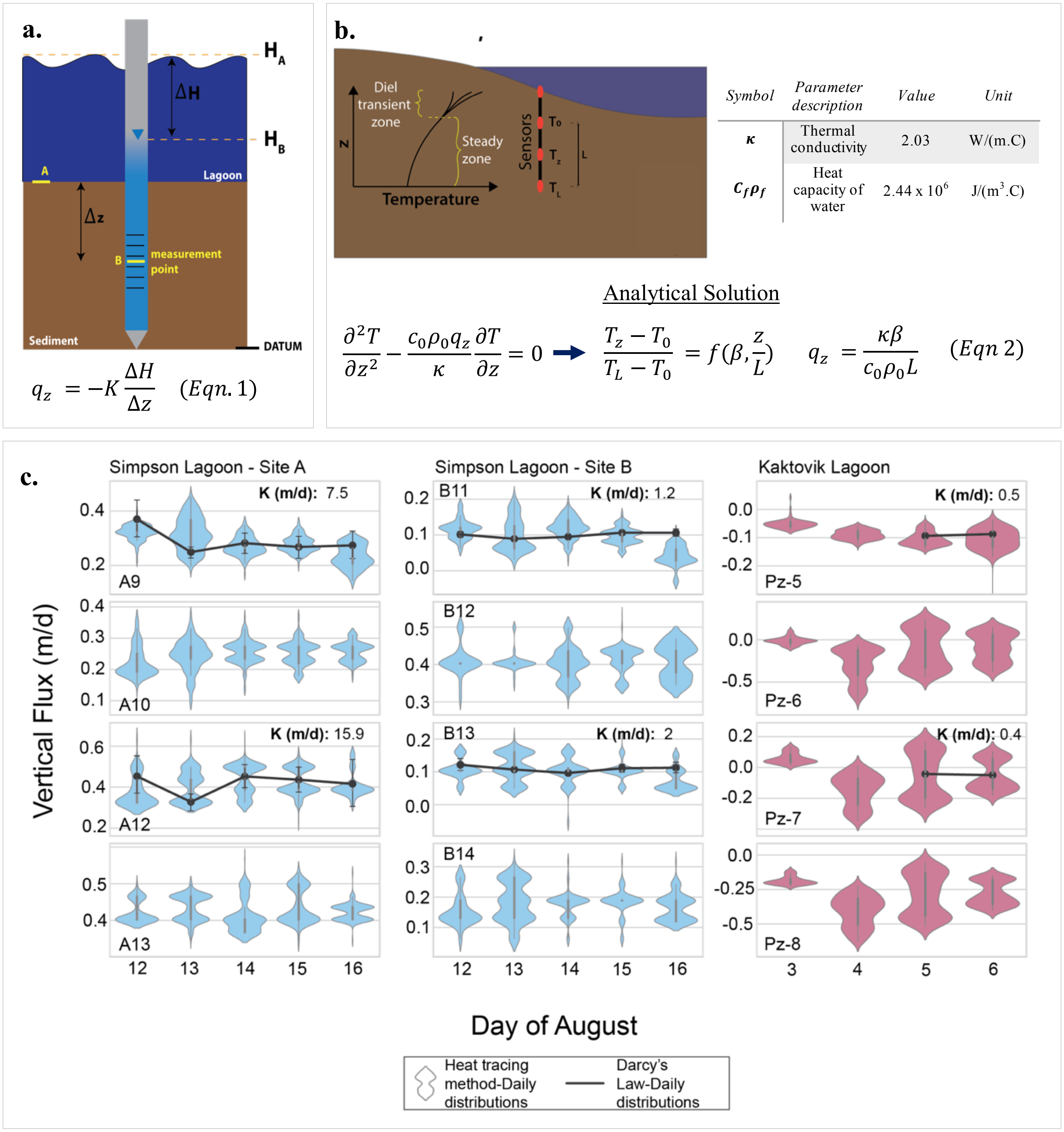

Figure S3. Field-based (in situ) methods used in the calculation of field base groundwater flux estimations and hydraulic conductivity values. Hydraulic conductivity, K, is estimated via Darcy's Law (Eqn 1, **(a)**) by equating vertical groundwater fluxes, q_z, with the ones found with Bredehoeft and Papadopulos (1965) method (Eqn 2, **(b)**). q_z vs time plots of each method were visually fitted by adjusting the K value **(c)**. (($\Delta H/\Delta L$: hydraulic gradient, T: temperature measured at different depths, κ: Thermal conductivity of solid-fluid complex, c_0: specific heat of fluid, ρ_0: density of fluid, β: type curve parameter.

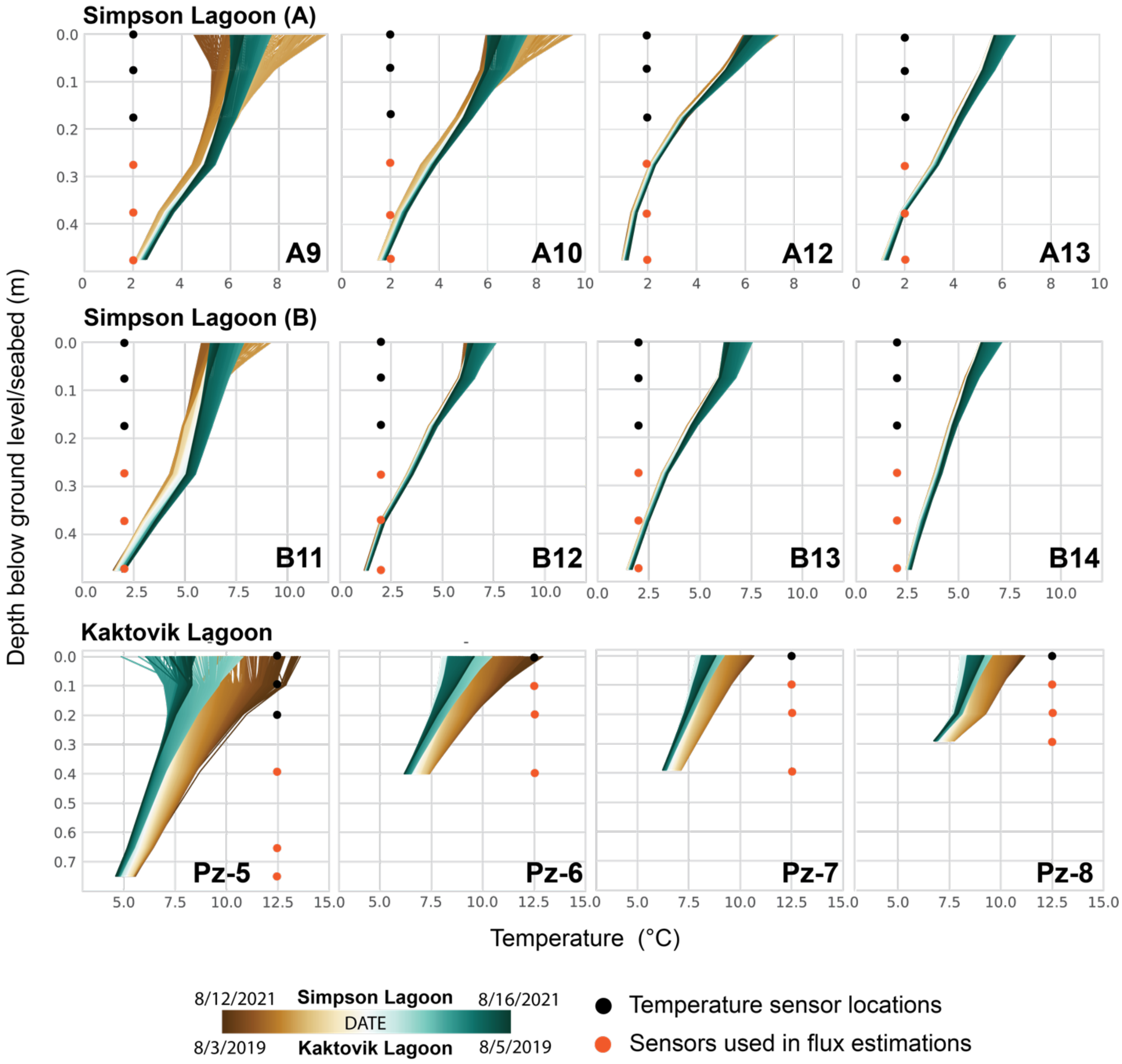

Figure S4. In situ measured time dependent vertical temperature profiles which were used to estimate vertical groundwater (Darcy) fluxes to Simpson Lagoon at Site A, Site B, and Kaktovik Lagoon. The dynamic diel zone is deeper and the temperature range is higher landwards in Simpson Lagoon (from A13 to A9, B14 to B11). In Kaktovik Lagoon, the decrease in vertical-temporal temperature variation in the seaward direction (from Pz-5 to Pz-8) is more moderate than that of Simpson Lagoon sites within similar lateral distances.

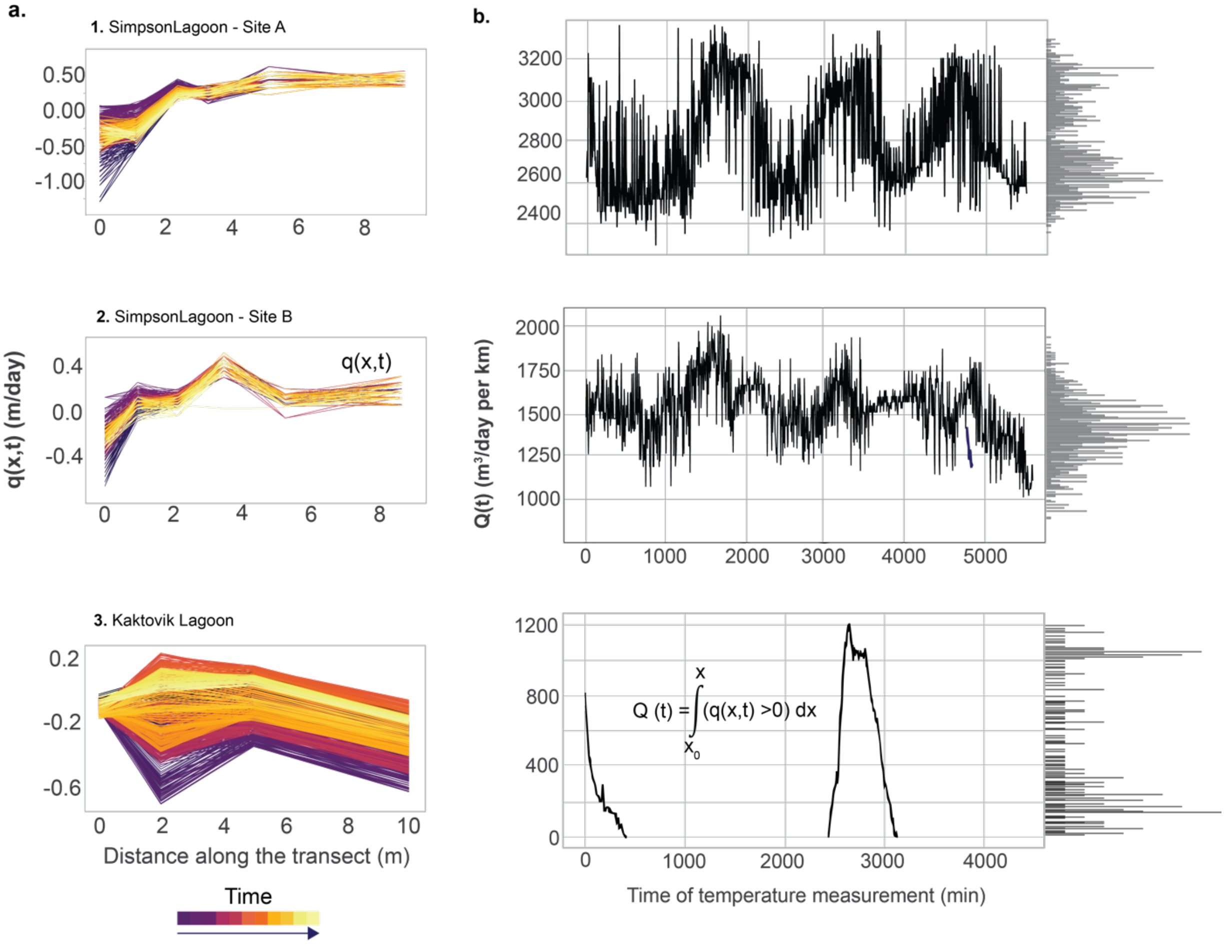

Figure S5. In situ method estimates of vertical groundwater fluxes: (a) as a function of x and t, m.day^{-1} (b) integrated flowrate Q as a function of t, m^3.day^{-1}.km^{-1}. The fresh groundwater discharge to the lagoons were calculated by integrating the fluxes over a seepage area. Here we summarize how we implemented the integration for the fluxes calculated via heat trancing method. First, the groundwater flow rate (m.day^{-1}) vs distance along piezometer transects were plotted for each time step **(a)**. Then, the positive areas under the curve for each time step was computed to estimate the groundwater input to the lagoons (Q, m^3.day^{-1}.km^{-1}) **(b)**. Histograms of Q are given on the right side of each time-series plot in (b).

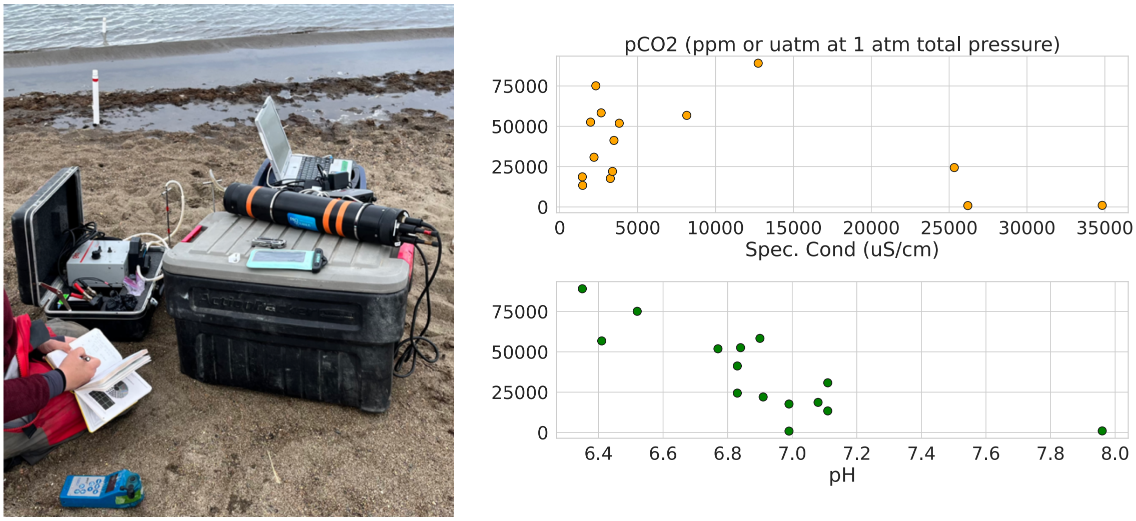

Figure S6. P_{CO2} measurements. (Left) Our in-situ setup; peri-pump is on the left and P_{CO2} sensor is on the right of the picture. Groundwater was pumped from stationary PVC piezometers and portable aluminum mini-point samplers (both shown in picture) at depths between 50-70 cm (up to 90 cm in the inter-tidal area). (Right) Measured P_{CO2} (μatm) with respect to sample salinity (given as specific conductance, $\mu S.cm^{-1}$) and pH.

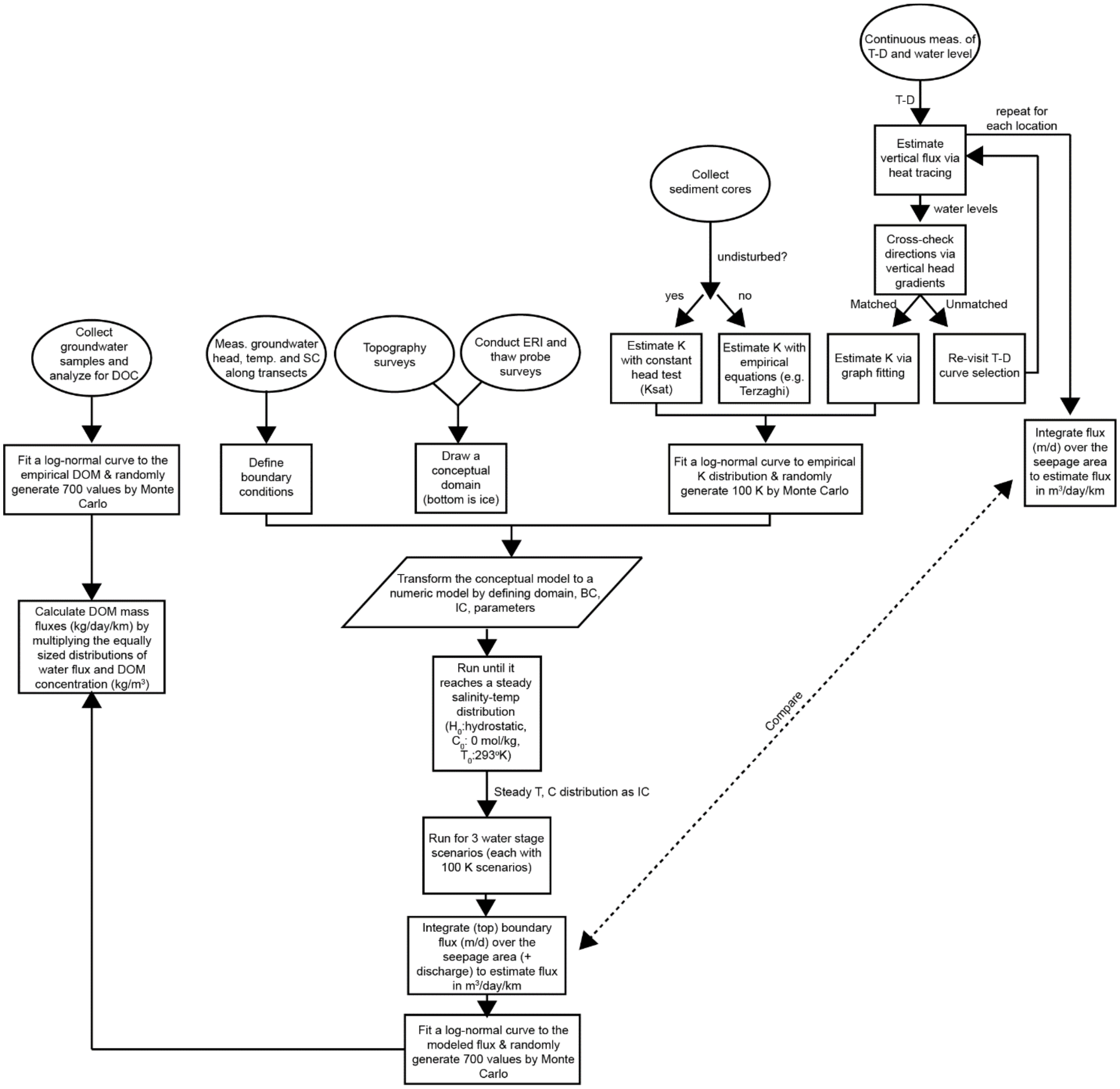

Figure S7. Diagram of the workflow for estimating fresh submarine groundwater discharge via numerical modeling and in situ observational techniques

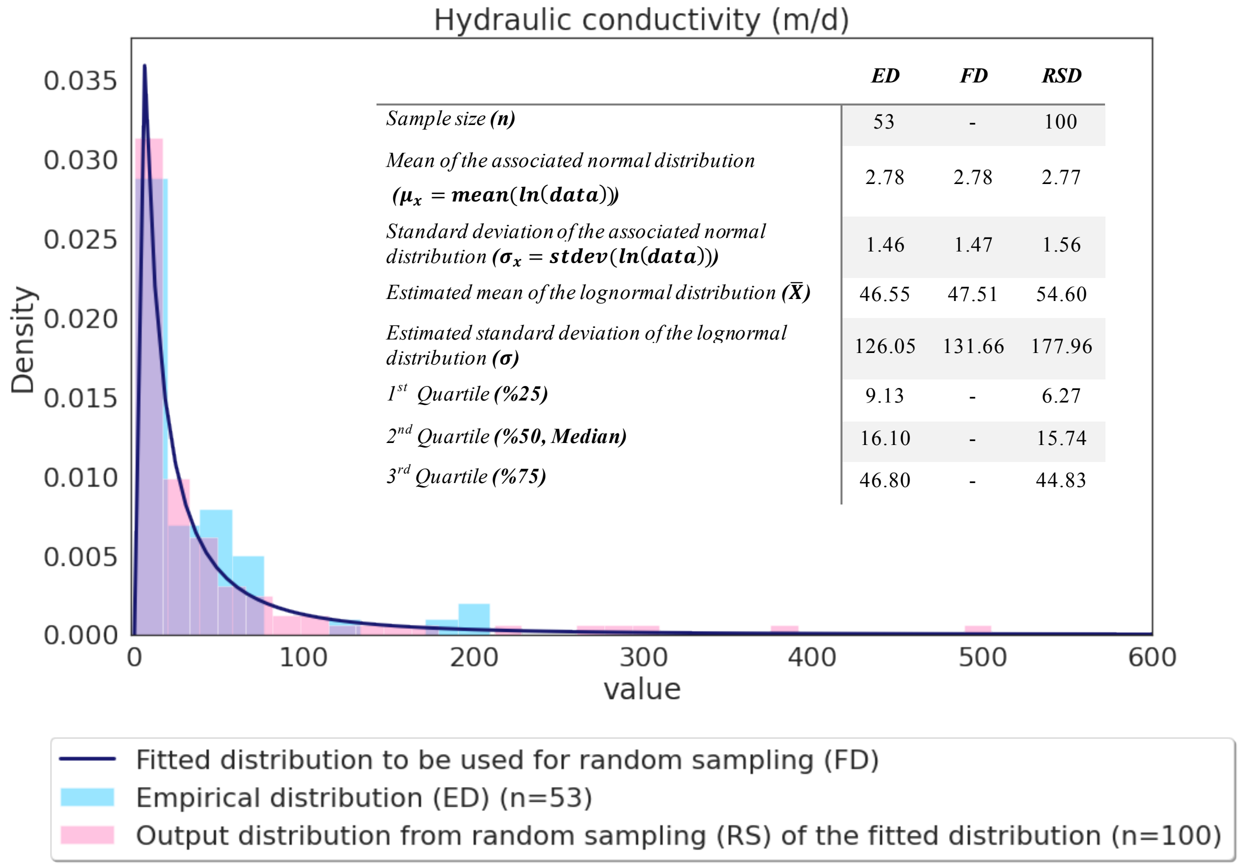

Figure S8. Comparison of the empirical distribution of hydraulic conductivity values with fitted probability density function and the randomly sampled distribution. Estimated hydraulic conductivity (K) values for Kaktovik and Simpson Lagoon were merged to create an empirical distribution (ED, n=53) and fitted with a lognormal distribution (FD). We randomly sampled (RS, n=100) the fitted distribution and ran numerical models in a parametric sweep mode for 100 different K values representing the empirical distribution. Descriptive statistics of the distributions are given in the tabular inset.

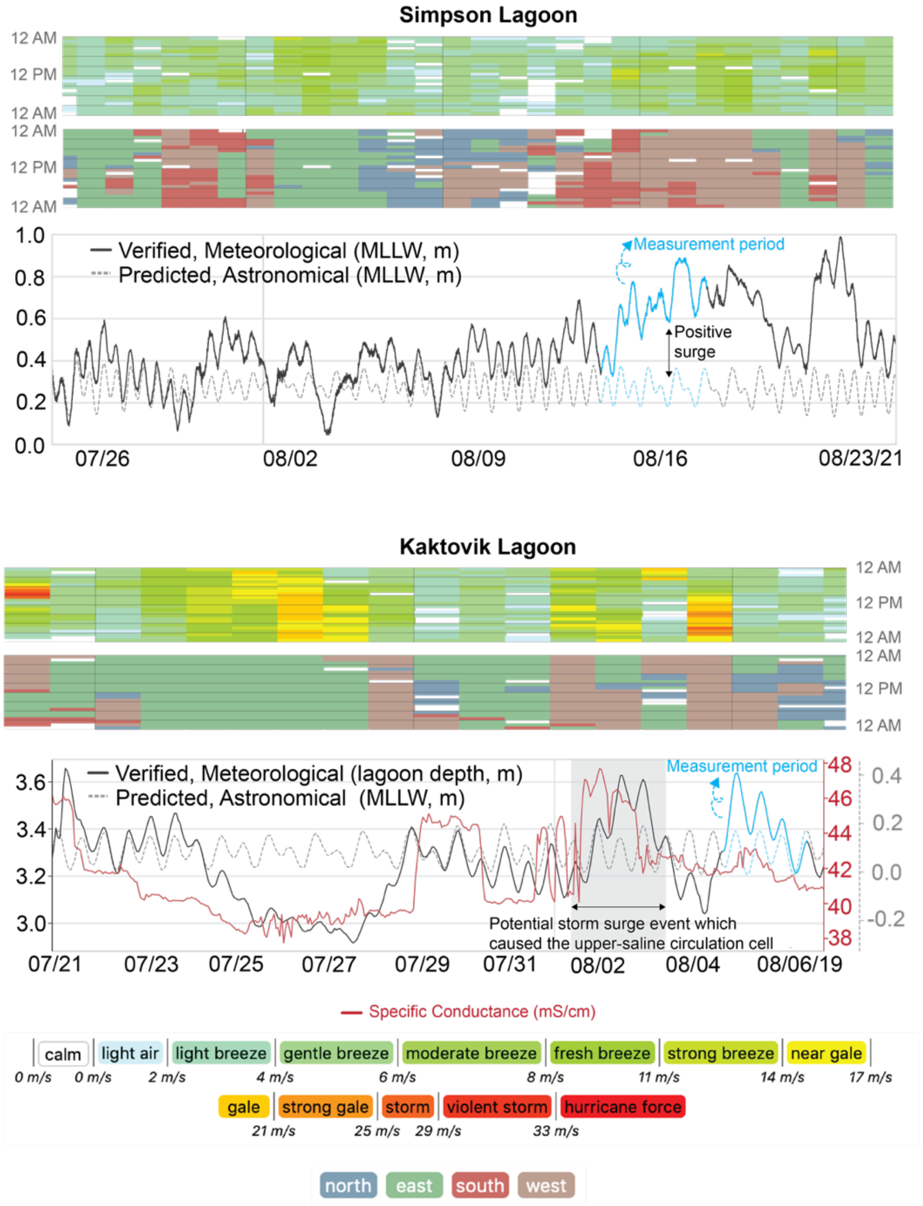

Figure S9. Lagoon level and salinity fluctuations covering several days before our measurement period (marked with blue). Both Simpson and Kaktovik Lagoons experienced positive surge during our monitoring period (SL, Aug 15, 2021) or night before (KL, Aug 4, 2019), respectively. Gray box in KL shows the period of potential storm surge which may have caused the upper-saline circulation cell mentioned in the main text and indicated in Figures 2a and 3a. Wind and wind direction: © WeatherSpark.com (Prudhoe Bay); SL level: NOAA (Site: 9497645); KL level/salinity: BLE-LTER (Beaufort Lagoon Ecosystems LTER, 2020). MLLW: Mean Lower Low Water, the average level of the lowest tide for each day computed over a 19-year period. *Predicted: astronomical, Verified: measured meteorological.*

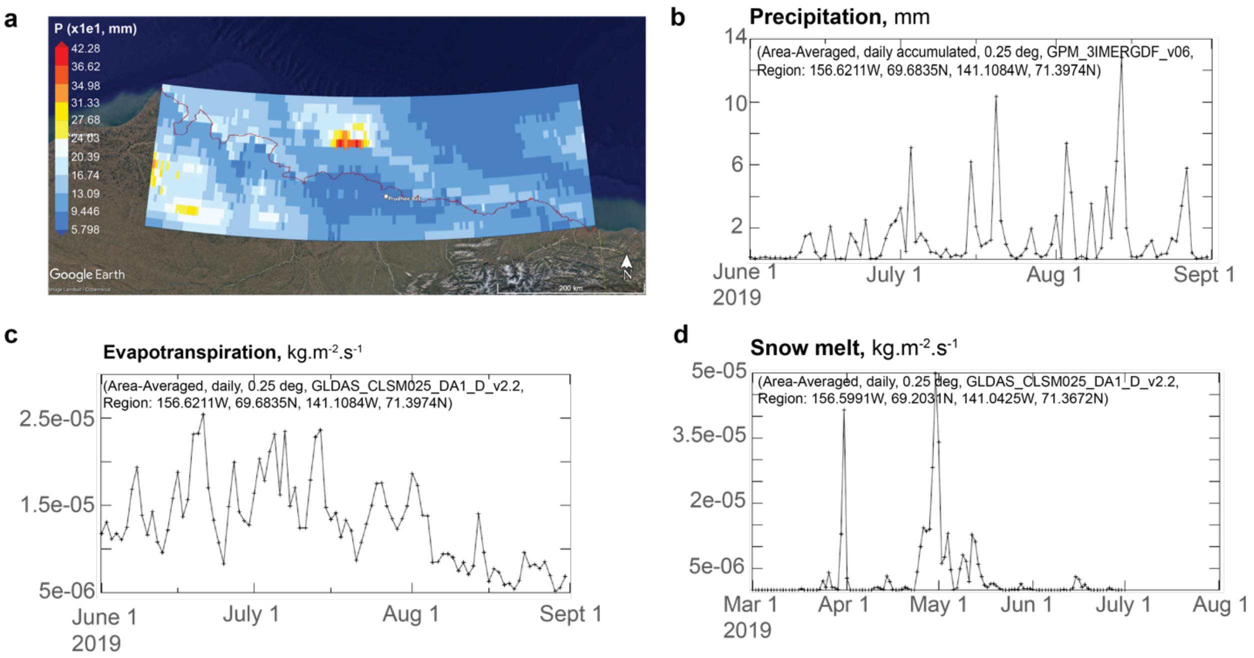

Figure S10. (a) Accumulated daily precipitation (mm) over 06-01 to 08-31-2019. Product of GPM IMERG Final Precipitation L3 1 day 0.1 degree × 0.1 degree V06. Red colored area along the coastline is the selected contributing area of ~200 m in width. The accumulated precipitation over the June-September period within the coastal zone ranges between 58 and 204 mm along the Beaufort Sea coast of Alaska. (b) Area averaged (over the bounding-box in (a)) precipitation given as a time-series indicates that precipitation very minimal in early June and picks up after mid-June, peaking in mid-August. Area-averaged accumulated precipitation is 130 mm over 06-01 to 08-31-2019, which is higher than the long-term (2016-2020) average summer precipitation (83.9 mm, (Rawlins, 2021)). (c) Area averaged evapotranspiration (ET, mm) during June 1 – August 31, 2019 and b) snowmelt during March – July, 2019. The accumulated ET is calculated as 39 mm (density of water: 1000 $kg.m^{-3}$). Most of snowmelt occurs from April to May. The presented analyses and visualizations were produced with and copied from the Giovanni online data system, developed and maintained by the NASA GES DISC

Table S1. Parameters and settings used in the electrical resistivity data inversion. The lagoon bottom transect underwater resistivity was inverted with AGI EarthImager™ 2D software. The tundra and shore parallel transect inversions were completed with RES2DINV®

AGI EarthImager™ 2D: Lagoon bottom

Parameter	*Setting*
Inversion method	Smooth Model Inversion
Number of iterations	8
Max RMS Error %	3
Stop Criteria	L2 Norm
Number of CG Iterations	6
Starting Iteration of Quasi Newton Method	20
Smoothness Factor	1000
Damping Factor	0.1
Estimated Noise (%)	3
Resolution Factor	0.2
Starting model	Average Apparent Resistivity
Min Resistivity	0.1
Max Resistivity	200
Model parameter width-height	4-1
Horizontal/Vertical Roughness Ratio	0.1

Table S1, continued. Parameters and settings used in the electrical resistivity data inversion. The lagoon bottom transect underwater resistivity was inverted with AGI EarthImager™ 2D software. The tundra and shore parallel transect inversions were completed with RES2DINV®.

RES2DINV®: At the shore

Parameter	**Setting**	
	Shore Parallel	Tundra
Inversion method	Robust inversion (L1)	Robust inversion (L1)
Robust data constraint cutoff factor	0.05	0.05
Method to solve the least-square equations	Incomplete Gauss-Newton	Incomplete Gauss-Newton
Convergence limit for Incomplete Gauss-Newton method	0.005	0.005
Ratio of first layer thickness to the unit electrode spacing	0.5547	0.5547
Rate at which the layer thickness increases with depth	1.1	1.1
Actual electrode spacing (m)	1.5	1
Model refinement	Model cells with widths of half the unit spacing (0.75m)	Model cells with widths of half the unit spacing (0.5m)
Damping factor	0.15	0.2
Vertical-horizontal flatness ratio	1	1
% change in RMS error for convergence	5	5
Number of iterations	7	7
Limit resistivities	None	0.02 – 5000 Ohm-m

Table S2. Equations solved in numerical models

Description	Equation
Groundwater flow (Eqn 3)	$S_p \rho_f \frac{\partial p}{\partial t} + \nabla \cdot \left(\rho_f u\right) = 0$<br>$u = -\frac{k}{\mu}(\nabla p + \rho_f g)$ **Darcy flux**<br>$p: pore\ pressure, S_p: storage\ coefficient, \rho_f: water\ density,$<br>$g: gravitational\ accelaration, \mu: fluid\ dynamic\ viscosity, k: permeability$
Storage (Eqn 4)	$S_p = \phi X_f + (1-\phi)X_p$<br>$X_f: fluid\ compressibility, X_p: effective\ matrix\ compressibilty, \phi: porosity$
Salt transport (Eqn 5)	$\frac{\partial(\phi c_i)}{\partial t} + \nabla \cdot J_i + u \cdot \nabla c_i = 0\ ;\quad c_i: Salt\ concentration$<br>$J_i = -\left(D_{D,i} + D_{e,i}\right)\nabla c_i\ ;\quad Dispersive\ flux$<br>$D_{e,i} = \phi^{4/3} D_f\ ;\ Effective\ diffusion\ coefficient$<br>$D_{D,i} = D_{D,i}(\alpha_L, \alpha_T)\ ;\quad Dispersion\ tensor$
Heat transport in porous media (Eqn 6)	$\left(\rho_f C_{p,f}\right)_{eff} \frac{\partial T}{\partial t} + \rho_f C_{p,f} u \cdot \nabla T + \nabla \cdot q = 0\ ;\quad T: temperature$<br>$q = -k_{eff} \nabla T\ ;\quad Conductive\ heat\ flux$<br>$\left(\rho_f C_{p,f}\right)_{eff} = (1-\theta_s)\rho_f C_{p,f} + \theta_s \rho_s C_{p,s}\ ;\quad C_{p,f}: fluid\ heat\ capacity$<br>$k_{eff} = (1-\theta_s)k_f + \theta_s k_s + k_{disp}\ ;\ Effective\ thermal\ conductivity$<br>$k_s = \frac{k_b}{\theta_s}\ (k\ of\ solids), \rho_s = \frac{\rho_b}{\theta_s}(\rho\ of\ solids), C_{p,s} = \frac{C_{p,b}}{\theta_s}(C_p\ of\ solids)$<br>$k_{dispersion} = k_{dispersion}(\lambda, \rho_f C_{p,f}, n)$
Water Density (Eqn 7)	$\rho_f = (999.83 + 0.808 * c_i - 0.0708 * (1 + 0.068 * T) - 0.003 * (1 - 0.012T)(35 - c_i) * T$
No flow/No flux/thermal insulation boundary (symmetry) (Eqn 8)	$-n \cdot \rho_f u = 0$<br>$-n \cdot J_i = 0$<br>$-n \cdot q = 0$

Table S3. Parameters used in numerical models

Symbol	Parameter description	Value	Unit
$\boldsymbol{C_0}$ *	Fresh groundwater concentration	0.49	kg.m^{-3}
$\boldsymbol{C_s}$ *	Lagoon water concentration	35	kg.m^{-3}
$\boldsymbol{\phi}$	porosity	0.4	
$\boldsymbol{\alpha_L}$	Longitudinal dispersivity	0.25	m
$\boldsymbol{\alpha_T}$	Transverse dispersivity	0.025	m
$\boldsymbol{D_f}$	Fluid diffusion coefficient (Millington and Quirk model)	1 x 10^{-9}	m^2.s^{-1}
$\boldsymbol{\mu}$	Dynamic viscosity	10^{-3}	Pa.s
$\boldsymbol{k_f}$	Thermal conductivity of porewater	0.6	W.(m.K)$^{-1}$
$\boldsymbol{k_b}$	Dry bulk thermal conductivity	1.4	W.(m.K)$^{-1}$
$\boldsymbol{C_{p,f}}$	Heat capacity of porewater	4182	J.(kg.K)$^{-1}$
$\boldsymbol{C_{p,b}}$	Dry bulk heat capacity at constant pressure	2000	J.(kg.K)$^{-1}$
$\boldsymbol{\rho_b}$	Dry bulk density	1600	kg.m^{-3}
$\boldsymbol{X_f}$	Fluid compressibility	4 x 10^{-10}	Pa^{-1}
$\boldsymbol{X_p}$	Effective compressibility (matrix)	1 x 10^{-7}	Pa^{-1}
$\boldsymbol{\gamma}$	Ratio of specific heats (fluid)	1	
$\boldsymbol{\lambda_L}$	Longitudinal dispersivity (heat)	0.1	m
$\boldsymbol{\lambda_T}$	Transverse dispersivity (heat)	0.01	m

Table S4. Set of equations used in calculation of P_{CO2}, $CO_{2(aq)}$ and DIC.

Parameter/Concept	*Equations*
Partial pressure of CO_2 (pCO_2)	$pCO_2(\mu atm) = wetCO_2\ (ppm_v) * P_{wet}(atm)$ $assume\ hydrostatic\ (P_{hydro} = 0)$ $\&\ atmospheric\ pressure\ (P_{atm} = 1)\ at\ sealevel$: $P_{wet} = P_{atm} + P_{hydro} = 1\ atm$; *total pressure of the internal gas including water vapor pressure*
Henry's Law and Fugacity (f) Corrections	$\left[CO_{2\ (aq)}^{*}\right] = K_0 * f(CO_2)$; $Aqueous\ CO_2\ concentration$ $f(CO_2) = pCO_2 * f$ $f = exp\left(\frac{P_{wet} * (B + 2 * x_2^2 * \delta)}{82.057 * T_{°K}}\right)$ $\delta = 57.7 - 0.118 * T_{°K}$ $x_2 = 1 - 10^{-6} * pCO_2$ $B = -1636.75 + 120408 * T_{°K} - 0.0327957 * T_{°K}^2 + 0.0000316528 * T_{°K}^3$
Carbonate chemistry equations	$[HCO_3^-] = \frac{K_1 * \left[CO_{2\ aq}^{*}\right]}{[H^+]}$ $[CO_3^{2-}] = \frac{K_2 * [HCO_3^-]}{[H^+]}$ $K_0, K_1, K_2\ are\ the\ equlibrium\ constants$
Dissolved Inorganic Carbon concentration	$DIC = \left[CO_{2\ (aq)}^{*}\right] + [HCO_3^-] + [CO_3^{2-}]$ *Assumption: TIC=DIC*

Table S5. Estimated fresh submarine groundwater discharge values (M: Median of the lognormal distribution, $\bar{X}$: Estimated mean of the lognormal distribution, σ: Estimated standard deviation of the lognormal distribution, IQR: Inter quartile range (%25 – %75), μ_x : mean normal distribution, σ_x : standard deviation of normal distribution)

		FSGD ($m^3.day^{-1}$ per km coastline)			
		**M**	**IQR**	$\bar{X}$	**σ**
Modeled	**Deep STE site:** Kaktovik Lagoon	5,788	2,331 – 16,776	20,457	69,345
	**Shallow STE site:** Simpson Lagoon	1,447	551 – 4,470	5,658	21,394
	**Beaufort Sea** coast of Alaska *($m^3.day^{-1}.km^{-1}$)*	2,894	983 – 9,595	13,682	63,226
	**Beaufort Sea** *($m^3.day^{-1}$ for 1,957 km coastline)*	5.7×10^6	$0.19 – 1.9 \times 10^7$	2.7×10^7	1.2×10^8
In situ	**Deep STE site**	319	170 - 859	584	896
	**Shallow STE site**	2,229	1,421 – 2,687	(μ_x) 2,085	(σ_x) 711
	**Beaufort Sea**	1,700	1,360 – 2,660	2,100	1,700

Table S6. Vertical salinity measurements in the seabed sediment. A piezometer was temporarily located on the transect between Pz4 and Pz5 in Kaktovik Lagoon to conduct a salinity profile survey (and sampling). s/d: shallow/deep piezometers. Salinity gradient: $[(T_{top}-T_{bottom})/|\text{Depth between measurement points}|]$

Site	Piezometer	Measurement Depth (cm, below seabed)	Specific Conductivity (μS.cm^{-1})	Temperature (°C)	Salinity Gradient (μS.cm^{-2})
Simpson Lagoon SITE A	A7s	17.6	23240	6.5	183.1
	A7d	62.7	14980	5.3	
	A8s	15.8	18920	8.1	153.9
	A8d	60.7	12010	4.6	
	A9s	17.7	20380	7.5	-26.0
	A9d	58.8	21450	5.8	
	A10s	22.4	26580	6.7	217.5
	A10d	47.5	21120	5.0	
	A11s	19.1	27250	6.5	-94.3
	A11d	72.0	32240	2.6	
	A12s	15.7	19830	8.3	-281.2
	A12d	49.8	29420	4.2	
Kaktovik Lagoon	Temporary-well	35	25600	7	465.5
	Temporary-well	55	16290	6.4	
	Temporary-well	75	17600	6.4	358.0
	Temporary-well	95	10440	4.9	
	Temporary-well	115	9321	4.9	81.3
	Temporary-well	135	7696	4.2	

Table S7. Estimated fresh submarine groundwater mass solute concentrations and fluxes. (*M*: Median of the lognormal distribution, $\bar{X}$: Estimated mean of the lognormal distribution, σ: Estimated standard deviation of the lognormal distribution, *IQR*: Inter quartile range (%25 – %75))

	M	$\bar{X}$	σ	IQR
	Kaktovik Lagoon			
C_{DOC} *(moles.m^{-3})*	2.92	3.1	1	2.75 – 3.25
C_{DON} *(moles.m^{-3})*	0.14	0.14	0.043	0.114 – 0.14
F_{DOC} *(moles.d^{-1}.km^{-1})*	18,750	68,167	238,167	6,667 – 49,833
F_{DON} *(moles.d^{-1}.km^{-1})*	714	2,357	7,571	214 – 2,000
	Simpson Lagoon			
C_{DOC} *(moles.m^{-3})*	5.58	5.75	1.5	4.7 – 7.25
C_{DON} *(moles.m^{-3})*	0.25	0.26	0.54	0.214 – 0.286
P_{CO2} *(μatm)*	32,872	38,527	23,550	19,497 – 52,409
C_{CO2} *(moles.m^{-3})*	1.57	1.8	1.0	0.98 – 2.40
C_{DIC} *(moles.m^{-3})*	7.42	8	2.9	5.25 – 9.9
F_{DOC} *(moles.d^{-1}.km^{-1})*	8,417	38,250	168,500	2,583 – 26,750
F_{DON} *(moles.d^{-1}.km^{-1})*	357	1,714	7,214	143 – 1,143
F_{CO2} *(moles.d^{-1}.km^{-1})*	2,388	12,244	61,583	725 – 8,304
F_{DIC} *(moles.d^{-1}.km^{-1})*	11,583	53,667	243,500	3,500 – 37,167
	Beaufort Sea coast of Alaska			
F_{DOC} *(moles.d^{-1}.km^{-1})*	9,667	47,917	232,250	2,833 – 33,750
F_{DON} *(moles.d^{-1}.km^{-1})*	429	2,214	10,714	143 – 1,500

Table S8. Field measured water parameters, P_{CO2} and estimated inorganic carbon concentrations

Sample ID	Groundwater #1	Groundwater #2	Groundwater #3	Groundwater #4	Groundwater #5	Groundwater #6	Groundwater #7	Groundwater #8	Groundwater #9	Groundwater #10	Groundwater #11	Groundwater #12	Groundwater #13	Lagoon #1	Lagoon #2
Specific conduc. (μ $S.cm^{-1}$)	2312	2656	12740	25330	3245	1974	8151	3477	3384	3818	1466	1447	2196	34830	26200
T (° C)	14.8*	17.7	15.2	12	12.4	15.5	11.7*	15	11.8	10.8	11.8	11.5	10.6	13.8	13
pH	6.52	6.9	6.35	6.83	6.99	6.84	6.41	6.83	6.91	6.77	7.11	7.08	7.11	7.96	6.99
pCO_2 (μ atm)	75127	58351	89111	24408	17651	52578	56777	41242	22016	51902	13372	18657	30801	974	835
CO_2 (aq) ($mol.m^{-3}$)	3.441	2.419	3.893	1.146	0.864	2.336	2.808	1.855	1.098	2.674	0.670	0.945	1.604	0.042	0.038
CO_3^{2-} ($x10^{-3} mol.m^{-3}$)	1.978	10.21	3.781	15.18	4.749	5.640	2.012	5.877	4.152	5.427	3.969	4.763	10.97	148.7	1.149
HCO_3^- ($mol.m^{-3}$)	5.889	10.80	6.370	6.268	4.386	8.341	4.390	6.974	4.607	8.099	3.968	5.176	9.724	3.546	0.310
DIC ($mol.m^{-3}$)	9.333	13.23	10.27	7.430	5.254	10.68	7.199	8.835	5.710	10.78	4.642	6.125	11.34	3.737	0.349
Alkalinity ($mol.m^{-3}$)	5.893	10.83	6.378	6.300	4.395	8.352	4.393	6.986	4.616	8.110	3.976	5.185	9.746	3.868	0.314

* average of sample temperatures collected on the same day used to replace a missing temperature measurement.

Table S9. Sensitivity of inorganic carbon concentrations to varying pH

		Groundwater													Lagoon	
Sample ID	*Δ pH*	*#1*	*#2*	*#3*	*#4*	*#5*	*#6*	*#7*	*#8*	*#9*	*#10*	*#11*	*#12*	*#13*	*#1*	*#2*
CO_3^{2-} ($x10^{-3} mol.m^{-3}$)	−0.05	1.571	8.108	3.003	12.06	3.773	4.480	1.598	4.668	3.298	4.311	3.153	3.784	8.714	118.1	0.913
	0	1.978	10.21	3.781	15.18	4.749	5.640	2.012	5.877	4.152	5.427	3.969	4.763	10.97	148.7	1.149
	+0.05	2.490	12.85	4.760	19.11	5.979	7.100	2.533	7.399	5.228	6.833	4.997	5.997	13.81	187.1	1.446
HCO_3^- ($mol.m^{-3}$)	−0.05	5.249	9.630	5.678	5.587	3.909	7.434	3.912	6.216	4.106	7.219	3.536	4.613	8.666	3.160	0.276
	0	5.889	10.80	6.370	6.268	4.386	8.341	4.390	6.974	4.607	8.099	3.968	5.176	9.724	3.546	0.310
	+0.05	6.608	12.12	7.148	7.033	4.921	9.358	4.925	7.825	5.170	9.087	4.452	5.807	10.91	3.979	0.348
DIC ($mol.m^{-3}$)	−0.05	8.692	12.06	9.574	6.745	4.776	9.774	6.722	8.075	5.208	9.897	4.210	5.561	10.28	3.321	0.315
	0	9.333	13.23	10.27	7.430	5.254	10.68	7.199	8.835	5.710	10.78	4.642	6.125	11.34	3.737	0.349
	+0.05	10.05	14.55	11.05	8.198	5.790	11.70	7.736	9.687	6.273	11.77	5.127	6.757	12.53	4.208	0.387
Alkalinity ($mol.m^{-3}$)	−0.05	5.252	9.646	5.683	5.612	3.916	7.442	3.915	6.225	4.113	7.227	3.543	4.620	8.684	3.419	0.280
	0	5.893	10.83	6.378	6.300	4.395	8.352	4.393	6.986	4.616	8.110	3.976	5.185	9.746	3.868	0.314
	+0.05	6.613	12.15	7.157	7.073	4.933	9.373	4.930	7.840	5.180	9.101	4.462	5.819	10.94	4.380	0.353

Table S10. Calculation of riverine DOC/N mass fluxes. Late summer riverine fluxes of DOC and DON referred in the paper to compare FSGD derived fluxes were estimated by using the data and the information provided in McClelland et al. (2014). Beaufort Sea coastline is 1957 km long.

	Sagavanirktok	*Kuparuk*	*Colville*	*Total*
Annual discharge (km^3/yr)	0.9 – 2.5	0.6 – 1.6	12.2 – 27.7	
Percent of annual discharge occurring in late summer (July–September, ~60days)	35%	37%	38%	
Late summer discharge (m^3/day)	(5.25 – 14.6) x 10^6	(3.67 – 9.86) x 10^6	(7.73 – 17.5) x 10^7	(8.62 –19.95) x 10^7
Late summer DOC concentration (kg/m^3)	1.4 x 10^{-3} ± 3.35 x 10^{-4}	5.14 x 10^{-3} ± 1.02 x 10^{-3}	3.7 x 10^{-3} ± 8.07 x 10^{-4}	
Late summer DON concentration (kg/m^3)	1.38 x 10^{-4} ± 2.94 x 10^{-5}	2.38 x 10^{-4} ± 2.1 x 10^{-5}	1.64 x 10^{-4} ± 6.72 x 10^{-5}	
Late summer DOC mass flux (kg/day)	7350 - 20440 ± 1758 - ± 4891	18863.8 – 50680.4 ± 3742.4 - ± 10057	286010 - 647500 ± 62382 - ± 141225	(3.12– 7.08) x 10^5 ± 6.78 x 10^4 – ± 1.56 x 10^5
Late summer DON mass flux (kg/day)	724.5 – 2014.8 ± 154.35 - ± 429.24	873.46 – 2346.7 ± 77 – ± 207	12677 - 28700 ± 5194.56 - ± 11760	(1.43– 3.3) x 10^4 ± 5.4 x 10^3 – ± 1.24 x 10^4
Late summer DOC mass flux (kg/day per km)	-	-	-	159.6 – 368.1 ± 34.7 – ± 79.9
Late summer DON mass flux (kg/day per km)				7.3 – 16.9 ± 2.8 – ± 6.3

Table S11. Estimated submarine groundwater discharge (SGD) to major seas and lagoons around the world

Study Area	*Method*	*Coastline length (km)*	*Total SGD (m^3/ day)*	*Total SGD (m^3/day per km)*	*Fresh SGD (m^3/day per km)*	*REF*
Beaufort Sea^{SupP}	Observation based numerical models	1957	$4.6x10^7$ – $6x10^8$	$2.4x10^4$ – $3.1x10^5$ (1–13% FSGD)*	3,063 (median, summer period, July+ August)	This study
Mediterranean Sea	Radium isotopes, Hydrologic approaches	46000	8.2×10^8 – 1.3×10^{10}	$1.8x10^4$ - $2.8x10^5$	4,169	(Kolker et al., 2021; Rodellas et al., 2015; Zektser & Dzhamalov, 2007)
Black Sea	Hydrologic mass balance	4340	4.4×10^7 **	1×10^4		(Zektser & Dzhamalov, 2007)
Buor Khaya Gulf, BKG (Laptev Sea, LS)^{SubP}	Radium isotopes	BKG : 392 LS:7500	BKG: 1.7×10^6 ** LS: $3.2x10^7$	$4.3x10^3$	43 – 559 (1–13% FSGD)*	(Charkin et al., 2017)
Simpson Lagoon^{SupP}	Observation based numerical models	69	8.9×10^5– 1.2×10^7	$1.3x10^4$ – $1.7x10^5$ (1–13% FSGD)*	1,691 (median, summer period, July+ August)	This study
Kaktovik Lagoon^{SupP}	Observation based numerical models	8	$3.4x10^5$ – 4.4×10^6	$4.3x10^4$ – $5.5x10^5$ (1–13% FSGD)*	5,545 (median, summer period, July+ August)	This study
Kaktovik Lagoon^{SupP}	Radon mass balance		8.6×10^5	$4.26x10^4$	400–2,100 (1–5% of total)	(Connolly et al., 2020)
Elson Lagoon	Radium isotopes(Lecher et al., 2016) and hydrologic mass balance(Rawlins, 2021)	34	$4x10^5$	$1.2x10^4$ (Lecher et al., 2016)	550 (Rawlins, 2021) (late summer to fall period, Aug+ Sept+ Oct period, averaged over 2016–2020)	(Rawlins, 2021)(Lecher et al., 2016)
Chilika lagoon, India	$^{87}Sr/^{86}Sr$	65	1.51×10^6	$2.3x10^4$		(Danish et al., 2020)
Little Lagoon, Alabama	Radon Mass balance	16	$5.2–25 \times 10^4$	$3.2–15.6 \times 10^3$		(Su et al., 2014)
Patos Lagoon, Brazil	Radium isotopes	240	8.5×10^7	3.5×10^5		(Niencheski et al., 2007)
Laoye Lagoon, Hainan, China	Radium isotopes	30	4.1×10^6	1.4×10^5		(Ji et al., 2013)
Indian River Lagoon, Florida	seepage meters, Cl– concentrations			1.2×10^5	20 - 900	(Martin et al., 2007)

* Estimated according to the assumption that FSGD= TSGD x (1–13%)

** Provided in the original study

^{SupP}: calculated for supra-permafrost, ^{SubP}: calculated for sub-permafrost
